# Thymoma‐associated autoimmune encephalitis with myasthenia gravis: Case series and literature review

**DOI:** 10.1111/cns.14568

**Published:** 2024-02-07

**Authors:** Miao Su, Qiuyan Luo, Zichao Wu, Huiyu Feng, Hongyan Zhou

**Affiliations:** ^1^ Department of Neurology The First Affiliated Hospital, Sun Yat‐sen University Guangzhou China; ^2^ Department of Neurology Guangzhou Women and Children's Medical Center Guangzhou China

**Keywords:** autoimmune encephalitis, myasthenia gravis, thymectomy, thymoma

## Abstract

**Objectives:**

This comprehensive review aimed to compile cases of patients with thymoma diagnosed with both autoimmune encephalitis (AE) and myasthenia gravis (MG), and describe their clinical characteristics.

**Methods:**

Clinical records of 3 AE patients in the first affiliated hospital of Sun Yat‐sen University were reviewed. All of them were diagnosed with AE between 1 November 2021 and 1 March 2022, and clinical evidence about thymoma and MG was found. All published case reports were searched for comprehensive literature from January 1990 to June 2022.

**Results:**

A total of 18 cases diagnosed with thymoma‐associated autoimmune encephalitis (TAAE) and thymoma‐associated myasthenia gravis (TAMG) were included in this complication, wherein 3 cases were in the first affiliated hospital of Sun Yat‐sen University and the other 15 were published case reports. 5/18 patients had alpha‐amino‐3‐hydroxy‐5‐methyl‐4‐isoxazolepropionic acid receptor antibody (AMPAR‐Ab) in their serum and cerebrospinal fluid (CSF). All of them had positive anti‐acetylcholine receptor antibody (AChR‐Ab). And 12/18 patients showed a positive response to thymectomy and immunotherapy. Besides, thymoma recurrences were detected because of AE onset. And the shortest interval between operation and AE onset was 2 years in patients with thymoma recurrence.

**Conclusions:**

There was no significant difference in the clinical manifestations between these patients and others with only TAMG or TAAE. TAAE was commonly associated with AMPAR2‐Ab. Significantly, AE more commonly heralded thymoma recurrences than MG onset. And the intervals of thymectomy and MG or AE onset had different meanings for thymoma recurrence and prognoses of patients.

## Graphical abstract

Thymoma induces the occurrence of autoreactive T cells, which activate humoral immunity and cause further MG and AE. After thymectomy, MG and AE may occur associated with thymoma recurrence, while post‐thymectomy MG can occur without thymoma recurrence but with long‐lived plasma cells and long‐memory T lymphocytes. (The graphic abstract was partly generated using Servier Medical Art by Servier, licensed under a Creative Commons Attribution 3.0 Unported License.)

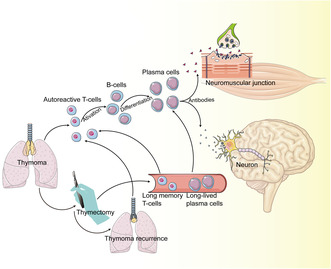

## 1 INTRODUCTION

Thymoma can occur with autoimmune disorders since it originates from epithelial cells of the thymus, the important immune organ for the maturation and differentiation of T‐lymphocytes.^1^ And the most common thymoma‐associated autoimmune disease is myasthenia gravis (MG), which occurs in 30% of patients with thymoma.^2^ MG is characterized by muscle weakness and fatigability due to the antibodies against autoantigens at the neuromuscular junction (NMJ). And while the main autoantibodies of thymoma‐associated MG (TAMG) against skeletal muscle are anti‐acetylcholine receptor antibodies (AChR‐Abs), the clinical manifestations have great heterogeneity.^2^

Thymoma‐associated autoimmune diseases also include autoimmune encephalitis (AE). But AE is an uncommon disease compared with MG according to a multicenter and retrospective study, in which only 1 patient had thymoma‐associated AE (TAAE) in 85 patients with thymoma.^3^ AE is a neurological disorder which presents with cerebral inflammation involving gray matter with or without white matter, caused by neuronal cell antibodies or intracellular antibodies.^4^ The most common antibody of thymoma‐associated AE was gamma‐aminobutyric acid receptor A antibody (GABA_A_R‐Ab), and alpha‐amino‐3‐hydroxy‐5‐methyl‐4‐isoxazolepropionic acid receptor antibody (AMPAR‐Ab) secondly.^5^

Previous series^5^, ^6^ of thymoma‐associated autoimmune diseases usually focused on the analysis of clinical features and autoantibody repertoire in patients with only thymomatous MG or thymomatous AE. Moreover, there were only some case reports for the clinical manifestations of patients with overlapping of TAMG and TAAE, which lacked systematic description. The objective of this article was to describe the sequence of MG and AE, the clinical presentations, the examination results and the management, as well as the prognosis of 3 patients with TAAE and TAMG from our center and conduct a comprehensive literature review with another 15 cases identified from previous case reports and case series.

## 2 MATERIALS AND METHODS

### 2.1 Case series

The clinical details of three patients with TAAE and TAMG from our institution were reviewed, and case 2 was diagnosed with AE elsewhere. In all cases, the clinical presentation, including memory impairments, confusion and abnormal behaviors and so on, brain MRI, CT of the chest, CSF analysis and cytology, immunosuppressive therapy, surgery, pathological classification of thymomas, and prognosis were recorded.

### 2.2 Review of the literature

Search terms “thymoma,” “autoimmune encephalitis,” and “myasthenia gravis” identified 15 case reports through Embase, MEDLINE, and Scopus databases from January 1990 to June 2022. The references of articles which were identified by these search terms also were filtered for more relevant articles. The clinical presentation, brain MRI, autoimmune antibodies test, immunosuppressive therapy, types of surgery, pathological classification of thymomas, and prognosis in these case reports were recorded in Table 1 and analyzed in Tables S1, S2 and Figure S1 in the supplementary material.

**TABLE 1:** Clinical characteristics of 18 patients with TAAE and TAMG in English case reports.

References	Sex	Age at diagnosis of thymoma	Onset of MG	Onset of AE	Thymoma recurrence	Seizure	Other neurological symptoms and psychiatric symptoms of AE	Skeletal muscle weakness of MG						Abnormal signal of MRI	CSF	Antibodies relative to AE	Antibodies relative to MG	Pathology of thymoma	Immunotherapy after thymectomy	Prognosis	Other autoimmune diseases
								O	F	B	N	R	L								
Our Case 1 (2021)	M	47	3 years after thymectomy	3 years after thymectomy	MG and AE	No	Memory deterioration and confusion					+	+	Normal	Lymphocytic pleocytosis	AMPAR2‐Ab	Ach‐R‐Ab, RYR‐Ab, Titin‐Ab	B2	Corticosteroids and iv‐IgG	Death	Hashimoto's thyroiditis
Our Case 2 (2021)	F	35	Over 10 days after thymectomy	3.6 years after thymectomy	No	No	Memory deterioration	+		+		+	+	Normal	Normal	AMPAR2‐Ab	Ach‐R‐Ab, Titin‐Ab	B1	Corticosteroids and iv‐IgG	Remission	No
Our Case 3 (2020)	M	49	Before the diagnosis of thymoma	Before the diagnosis of thymoma	No	No	Agitation, persecutory delusion and violent behavior	+				+		Normal	Protein level elevation	GABA_B_R‐Ab, NMDAR‐Ab	Ach‐R‐Ab	Sclerosing thymoma	Corticosteroids, iv‐IgG, PE and tacrolimus	Death	Alopecia Areata (patchy alopecia)
Monstad et al. (2009)^9^	F	49	Before the diagnosis of thymoma	1 year after thymectomy	No	No	Memory deterioration and confusion	+					+	Bilateral hippocampus	Lymphocytic pleocytosis	CRMP5‐Ab	Ach‐R‐Ab	Lymphoepithelial thymoma	Corticosteroid, PE and azathioprine	Remission	No
Khella et al. (2007)^10^	F	41	Before the diagnosis of thymoma	Before the diagnosis of thymoma	No	No	Headache, dizziness, memory deterioration, hypersomnolence, nystagmus and gait ataxia	+		+	+			Right globus pallidus, substantia nigra, and both thalami	Lymphocytic pleocytosis	NA	Ach‐R‐Ab	Noninvasive spindle cell thymoma	Corticosteroids, iv‐IgG, PE and cyclophosphamide	Death	No
Aysal et al. (2013)^11^	M	43	Before the diagnosis of thymoma	Before the diagnosis of thymoma	No	SGTCs, CPS	No	+	+		+			Left temporoparietal, left insular lobes, and left frontal lobe	Normal	NA	Ach‐R‐Ab	AB	No	Remission	No
Miyazaki et al. (2012)^12^	M	46	Before the diagnosis of thymoma	4 years after thymectomy	AE	SGTCs	Aphasia, delirium and visual hallucinations	+						Both temporal lobe	Protein level elevation	VGKC‐Ab	Ach‐R‐Ab	B2	Corticosteroids and iv‐IgG	Severe cognitive impairment	No
Hor et al. (2018)^13^	M	69	Before the diagnosis of thymoma	1 year after MG onset without thymectomy	No	No	Memory deterioration, confusion, personality changes and myokymia	+		+		+		Both mesial temporal regions	Normal	LGI1‐Ab	Ach‐R‐Ab	AB	Corticosteroids and iv‐IgG	Remission	Nephrotic syndrome
Luo et al. (2019)^14^	F	48	Before the diagnosis of thymoma	2 years after thymectomy	AE	No	Memory deterioration, confusion, behavioral changes and orientation obstacle	+						Both mesial temporal regions and hippocampus	Normal	AMPAR2‐Ab	Ach‐R‐Ab	B2	Corticosteroids and azathioprine	Remission	No
Liu et al. (2018)^15^	F	19	Before the diagnosis of thymoma	1 month after thymectomy	No	CPS	Memory deterioration, aphasia, confusion, delusions and visual hallucinations	+		+				Left insular cortex and hippocampus	No	VGKC‐Ab	Ach‐R‐Ab	B2	Corticosteroids, PE and azathioprine	Remission	Systemic lupus erythematosus (SLE)
Shaulov et al. (2012)^8^	F	66	18 years after thymectomy	A few months after thymectomy	No	Grand mal seizure	Memory deterioration and confusion	+						Bilateral hippocampus	Normal	NA	Ach‐R‐Ab	B2	No	Remission	No
Li et al. (2015)^16^	F	47	8 months after AE onset without thymectomy	Before the diagnosis of thymoma	No	No	Apathy, aggressive behavior, aphasia, Memory deterioration and confusion	+		+				Normal	Normal	AMPAR2‐Ab	Ach‐R‐Ab	B1	Corticosteroids	Remission	No
Hammoud et al. (2009)^17^	F	43	Before the diagnosis of thymoma	4 years after thymectomy	AE	Seizure	Confusion and aphasic	+		+				Multifocal cortex and subcortex	Lymphocytic pleocytosis and protein level elevation	VGKC‐Ab	Ach‐R‐Ab	NA	Corticosteroids, iv‐IgG and mycophenolate mofetil	Death	No
Kodama et al. (1991)^7^	F	34	Before the diagnosis of thymoma	Before the diagnosis of thymoma	No	No	Memory deterioration, and disorientation	+		+				Left frontal lobe and both temporal lobe	Lymphocytic pleocytosis	NA	NA	NA	NA	Remission	No
Evoli et al. (1999)^18^	M	32	Before the diagnosis of thymoma	7 months after thymectomy	No	Grand mal seizure	Memory deterioration, and confusion	+					+	Both hippocampus	Lymphocytic pleocytosis	NA	Ach‐R‐Ab	Lymphoepithelial thymoma	Corticosteroids and PE	Remission	No
Buckey et al. (2001)^19^	F	47	Before the diagnosis of thymoma	7 years after thymectomy	AE	No	Memory deterioration, agitation and disorientation					+		Normal	Normal	VGKC‐Ab	NA	NA	PE	Death	No
Vernnino et al. (2002)^20^	M	34	Before the diagnosis of thymoma	1 month after thymectomy	No	Grand mal seizure	Memory deterioration, confusion and auditory hallucinations			+				Normal	Lymphocytic pleocytosis	ANNA1‐Ab	Ach‐R‐Ab	NA	Corticosteroids, PE and quetiapine	Remission	No
Vernnino et al. (2002)^20^	F	39	Before the diagnosis of thymoma	Before the diagnosis of thymoma	No	No	Personality change	+		+				Normal	No	ANNA1‐Ab, CRMP5‐Ab	Ach‐R‐Ab	NA	Corticosteroids and PE	Remission	No

Abbreviations: AE, autoimmune encephalitis; AMPAR2, Amino‐3‐hydroxy‐5‐methyl‐4‐isoxazolepropionic acid receptor 2; ANNA1‐Ab (or “anti‐Hu”), the type‐1 antineuronal nuclear antibody; B, bulbar muscles; CPS, complex partial seizures; CRMP5, Collapsin response mediator protein 5; CSF, cerebrospinal fluid; F, facial muscles; GABA _B_ R, gamma‐aminobutyric acid receptor B; iv‐IgG, immunoglobulin; L, limb muscles; LGI1, leucine‐rich, glioma inactivated 1; MG, myasthenia gravis; MRI, Magnetic resonance imaging; N, neck muscles; NA, data not available; NMDAR, anti‐N‐methyl‐D‐aspartate receptor antibody; O, ocular muscles; PE, plasma exchange; R, respiratory muscles; SGTCs, secondarily generalized tonic–clonic seizures; SPS, simple partial seizures.

## 3 RESULTS

### 3.1 Case series

#### 3.1.1 Case 1

**FIGURE 1:**
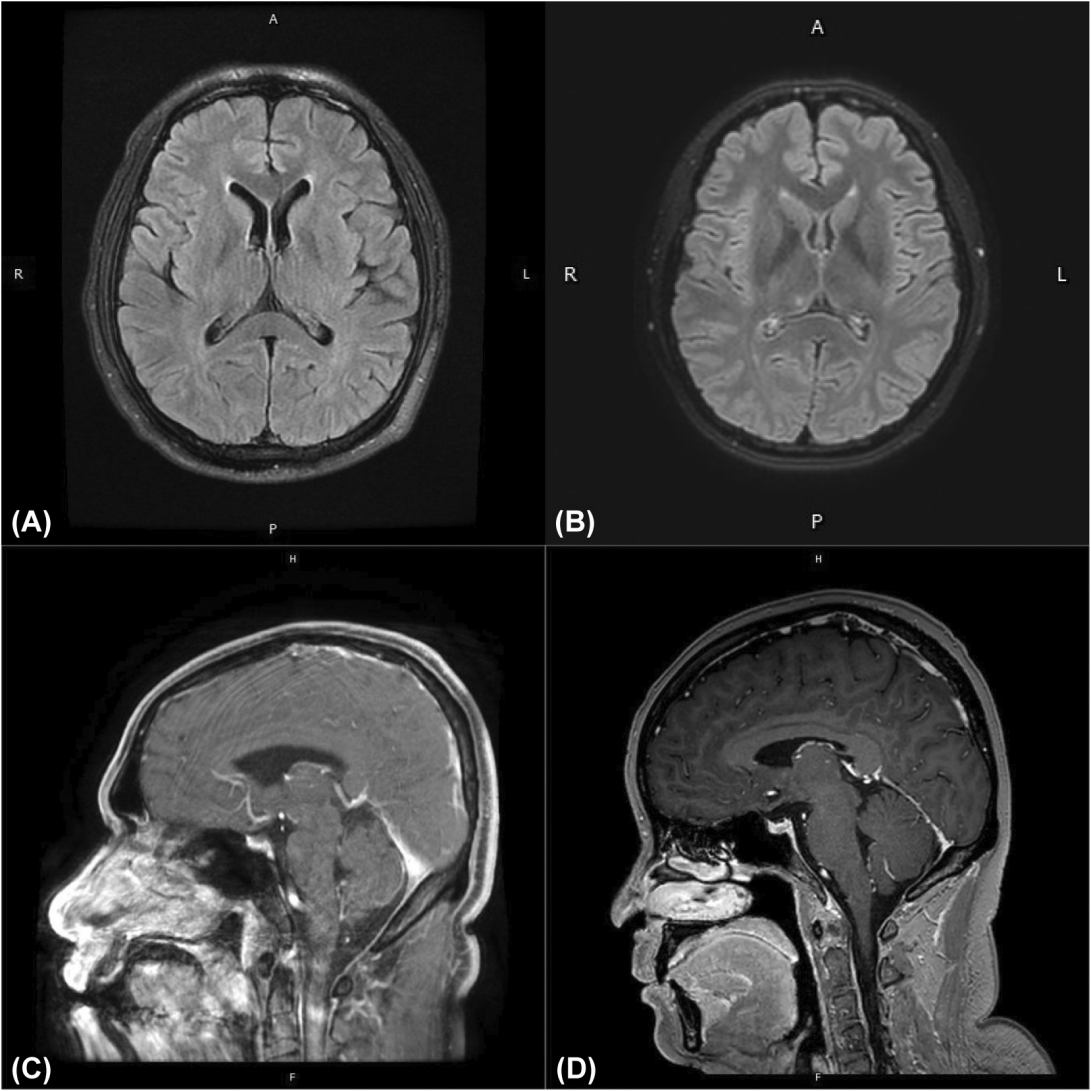
(A) T2‐FLAIR images of the brain MRI of patient 1 after AE onset showed no abnormal hyperintensity. (B) T2‐FLAIR images of the brain MRI of patient 2 after AE onset showed no abnormal hyperintensity. (C) Lack of enhancement of the brain MRI of patient 1 after AE onset. (D) Lack of enhancement of the brain MRI of patient 2 after AE onset. (Brain MRI images of patient 3 after AE onset cannot be accessed but its word report showed no abnormal hyperintense.) More images can be found in Figure S2.

A 50‐year‐old male accepted thymoma resection after thymoma detection in December 2018, and the histopathological classification was type B2 (World Health Organization thymoma classification). Before the thymectomy, he had no symptoms of MG or AE. But the patient had short‐term memory loss and confusion and became unresponsive after 3 years following thymectomy in December 2018. The disease progressed so rapidly that the patient could not walk due to the fatigable weakness of the bilateral leg, and had dysphagia and incontinence. The chest computed tomography (CT) revealed that the thymoma recurred, metastasized, and even invaded the pericardium. His brain MRI with Gd‐enhancement was normal (Figure 1A,1C). MG and AE were diagnosed concomitantly since serum antibody tests showed positive ACh‐R IgG (8.014 nmol/L), RyR (ryanodine receptor) IgG, Titin IgG, and AMPAR2‐Ab (1:32), while CSF AE antibody tests showed positive AMPAR2‐Ab (1:20). The pathogen Next Generation Sequencing (NGS) of Cerebrospinal Fluid (CSF) showed 3 human herpes virus type 4 (Epstein‐Barr virus, EBV) gene sequences. The other cerebrospinal fluid (CSF) examination was normal except for a lymphocytic pleocytosis of 41 cells/mm^3^ (normal: <5 cells/mm^3^). Besides, raised thyroid peroxidase antibodies (TPOAb) and thyroglobulin antibodies (TgAb), as well as decreased free thyroxine (fT4) and free triiodothyronine (fT3) were present with thyroid stimulating hormone (TSH) in the thyroid function assessment. The repetitive nerve stimulation (RNS) of electromyography and neostigmine test were unable to be completed due to the progressive symptoms of this patient.

Intravenous immunoglobulin (IVIg) 0.4 g/(kg*day) for 5 days and methylprednisolone 40 mg/day were given. But the symptoms did not improve after immunotherapy. On the 4th day after admission, he was in a light coma and a continued hypoxemia because of the disease progression and the pulmonary infection. An urgent trachea intubation was performed and the respirator was used to support his respirations. The thoracic surgeons and the radiation oncology physicians suggested that there was no indication for further surgery, chemotherapy, or radiotherapy. The patient was in a consistent coma and presented unstable blood circulation which needed norepinephrine to stabilize blood pressure. The family finally gave up and the patient died soon after discharge from day 14 after admission.

#### 3.1.2 Case 2

A 39‐year‐old woman underwent surgery following the diagnosis of thymoma and the histopathology showed type B1 thymoma. She developed generalized MG with fluctuated weakness, which included left ptosis, dysphagia, dysarthria, breathing disorders, and bilateral brachial weakness with swelling and pain over 10 days after the operation. Her muscle weakness was alleviated in the morning or after rest. And the patient had a positive ocular fatigue test and a good response to cholinesterase inhibitor therapy but a negative neostigmine test. Furthermore, her serum antibody test showed positive ACh‐R IgG. The electromyography showed myogenic damage, normal RNS, and an elevated serum muscle enzyme level. But she still had fatigable weakness after her serum muscle enzyme level declined to a normal level. the CT of the chest was normal without any thymoma recurrence or residual tumor. She was treated with methylprednisolone 40 mg/day and intravenous immunoglobulin (IVIg) 0.4 g/(kg*day) for 5 days. Then her symptoms of MG improved. She received radiotherapy after remission and continued taking methylprednisolone (25 mg once a day) and pyridostigmine bromide (60 mg 3 times a day) for MG.

The onset of AE occurred 3.6 years after the operation since the patient stopped taking methylprednisolone for 2 months. She experienced short‐term memory loss and repeatedly checked the time and place of her appointment with her friends. Besides, confusion and alopecia areata, which included localized hair loss of the head and pubis, occurred. MRI of the brain with Gd‐enhancement was normal (Figure 1B,1D). CT of the chest and PET imaging were performed and no signs of malignancy recurrence was suspected. The electroencephalogram (EEG) was normal. The serum antibodies testing showed positive AMPAR‐Ab (1:32). CSF revealed positive AMPAR‐Ab (1:32) and positive anti‐N‐methyl‐D‐aspartate receptor antibody (NMDAR‐Ab) (1:3.2). Normal in the CSF were the CSF pressure, white blood cell (WBC), protein, glucose, chloride, and bacteriological culture. She accepted IVIg 0.4 g/(kg*day) for 5 days and corticosteroid impulse therapy (methylprednisolone 500 mg/day for 3 days and follow‐up oral prednisone 50 mg/day). She was discharged since her memory was reversed.

Nine months later, the patient went back for further assessment. The serum antibody test revealed negative Ach‐R IgG and NMDAR‐Ab, but positive Titin IgG and AMPAR‐Ab (1:32). And she had lower AMPAR‐Ab (1:1) in CSF. Neuropsychological testing showed a mini‐mental state examination (MMSE) of 30/30. The testing related to MG showed a quantitative myasthenia gravis score (QMGS) of 2, a myasthenia gravis‐activities of daily living profile (MG‐ADL) of 100, and a modified Rankin Scale (mRS) of 1/5. Now, her daily activity is compatible with before.

#### 3.1.3 Case 3

A 49‐year‐old man presented to our neurological clinic in January 2022 with fluctuated bilateral ptosis, visual hallucination, and delusion of persecution. His ptosis was alleviated in the morning. CT of the chest showed an anterior superior mediastinal mass, considering the thymic tumor and left pleural metastasis. The serum antibodies testing showed positive GABA_B_R‐Ab (1:100). CSF revealed positive GABA_B_R‐Ab (1:100). His MRI of brain with Gd‐enhancement was normal. The neostigmine test was negative. MG's proof of diagnosis is a positive ACh‐R IgG serum test (14.57 nmol/L). The CSF analysis showed protein level elevation of 70 mg/dL (normal: <50 mg/dL). Normal investigations included CSF pressure, glucose, chloride, cell count, and bacteriological culture. No tumor cell was detected in CSF. The repetitive nerve stimulation (RNS) of electromyography was unable to be completed due to the progressive symptoms of this patient.

On the 1st day after admission, the patient was agitated and had violent and self‐injury behaviors. In a state of great agitation, he suddenly went into a coma, and then his carotid pulse disappeared, his nail bed and lips were cyanosed, and the oxygen saturation was too low to be measured. Chest compressions, ventilation with the balloon, and an intramuscular injection of epinephrine (1 mg) were given for emergency treatment and the patient's spontaneous respiration was restored in 3 minutes. After tracheal intubation, he accepted further treatment in the intensive care unit (ICU) with mechanical ventilation. The symptoms persisted despite increasing immunosuppressive medication including methylprednisolone impulsion 1 g/day for 3 days and subsequently 40 mg/day, tacrolimus 2.184 mg/day, intravenous immunoglobulin (IVIg) 0.4 g/(kg*day), and plasma exchanges for 5 days. The thymoma resection was performed on the 14th day after admission and the histopathology was sclerosing thymoma. Postoperative CSF GABA_B_R‐Ab titer decreased (1:1+), but serum GABA_B_R‐Ab remained (1:100) and psychiatric symptoms and muscle weakness were not improved. His family finally gave up and the patient died soon after discharge from day 22 after admission.

### 3.2 Literature review

Over the past 32 years, we identified 18 patients who had been diagnosed with thymoma, MG, and AE, including 15 cases that have been reported, and of them, 11 (61.11%) were female. MG and AE were diagnosed concomitantly in 9/18 patients (50%). The median age at thymoma diagnosis was 44.5 years, from age 19 to 69. The earliest case was reported by Kodama et al.^7^ in 1991, in which a 34‐year‐old woman was found to have a thymoma after presenting with AE and MG symptoms at the same time. Due to the limitation of detection technique, AE‐associated antibody was not detected in this patient. All the features of patients and their diseases are summarized in Table 1 and Table S1.

Our results showed that 10/18 patients (55.56%) had MG onset earlier than AE, and in these patients, the interval between MG and AE was from 1 month to 7 years with a mean interval of 2.335 years and a median interval of 1.5 year. 6/18 patients (33.33%) were diagnosed with MG and AE concomitantly, while 2/18 patients (11.11%) presented AE symptoms at first, including one patient with an interval of 18 years and the other patient with an interval of 8 months. In 18 patients, the most common skeletal muscle group affected by MG was ocular muscle (15/18, 83.33%), followed by bulbar muscle (9/18, 50%). The other muscle groups involved respiratory muscle (5/18, 27.78%), limb muscle (4/18, 22.22%), neck muscle (2/18, 11.11%), and facial muscle (1/18, 5.56%). The most common neurological symptoms in AE were memory loss (13/18, 72.22%) and confusion (10/18, 55.56%), followed by seizure (7/13, 38.89%). Only 4 patients with seizures had positive AE‐associated antibodies, among which VGKC‐AE was more common (3/7). 4/18 patients had another autoimmune disease, which included Hashimoto's thyroiditis (n = 1), alopecia areata (*n* = 1), nephrotic syndrome (*n* = 1), and systemic lupus erythematosus (*n* = 1).

At thymoma diagnosis with MG and AE, 16/18 patients (88.89%) had positive ACh‐R IgG in serum test. AE‐associated antibodies in serum/CSF test was AMPAR2‐Ab (5/18, 27.78%), VGKC‐Ab ((4/18, 22.22%)), ANNA‐1‐Ab (2/18, 11.11%), CRMP5‐Ab (2/18, 11.11%), LGI1‐Ab (1/18, 5.56%), GABA_B_R‐Ab (1/18, 5.56%), and data not available (5/18, 27.78%). Abnormalities of brain MRI occurred in 11/18 patients (61.11%), with temporal lobe and hippocampus involvement being the most common (9/11). All the 18 cases underwent thymoma resection and the WHO histological classification was B2 (5/18, 27.78%), AB (2/18, 11.11%), B1 (2/18, 11.11%), lymphoepithelial thymoma (2/18, 11.11%), sclerosing thymoma (1/18, 5.56%), noninvasive spindle cell thymoma (1/18, 5.56%), and the unclassified (5/18, 27.78%). The pathological type of case report from Shaulov et al.^8^ was classified as B2 since it was predominantly lymphocytic. No patient had type A or C. Only 1 patient had a complete remission after surgery and did not require immunosuppressive drugs, such as hormone therapy or immunosuppressive agents. 12/18 patients (66.67%) showed positive responses to thymectomy and immunotherapy and got clinical remission. Most of the patients were diagnosed with thymoma due to symptoms of MG or AE (15/18, 83.33%). The remaining 3 patients presented AE and MG symptoms after thymoma resection, including MG first (*n* = 1), AE first (*n* = 1), and simultaneity (*n* = 1). 16/18 patients (88.89%) had MG (4/18, 22.22%) or AE (12/18, 66.67%) onset preceding thymoma resection, including 5 patients with thymoma recurrences. All thymoma recurrences were detected because of AE onset. And the shortest interval between operation and AE onset was 2 years in patients with thymoma recurrence, whose histological classification was B2 (3/5) and the unclassified (2/5). Patients with thymoma recurrence or metastasis had the worst prognosis.

In the group of patients with poor outcome (patients did not get clinical remission after treatments 6/18 vs 12/18 of patients in the group with the good outcome), 3/6 patients were female, 3/6 patients had normal brain MRI after AE (vs 4/12), 4/6 patients had thymoma recurrence at AE onset (vs 1/12), and 5/6 finally died. The muscle groups affected by MG in patients with poor outcome included ocular muscle (4/6 vs 11/12), bulbar muscle (2/6 vs 7/12), respiratory muscle (3/6 vs 2/12), limb muscle (1/6 vs 3/12), neck muscle (1/6 vs 1/12), and facial muscle (0 vs 1/12). The most common neurological symptoms in AE in these patients were memory loss (2/6 vs 11/12), confusion (2/6 vs 8/12), and seizure (2/6 vs 5/12). The types of AE‐associated antibodies included 3/6 patients with VGKC‐Ab in patients with poor outcomes higher than patients with good outcomes (1/12), 1/6 AMPAR2‐Ab (vs 3/12), and 1/6 GABA_B_R‐Ab (vs 0/12). All of the patients underwent thymectomy and the multiple combinations of immunotherapies were more often used in patients with poor outcomes than with good outcomes. The comparison of two groups is summarized in Table S1 and Figure S1 in the supplementary material.

## 4 DISCUSSION

Cases in this study presented the clinical manifestations in patients diagnosed with thymoma with MG and AE and provided more information on these patients for clinical decisions. We found that the features of MG and AE in these patients were respectively similar to TAMG and TAAE. However, the intervals between thymectomy and MG or AE onset had different meanings for thymoma recurrence and prognosis of patients.

Our study found that patients diagnosed with thymoma with both MG and AE were most frequently associated with ocular muscle weakness, followed by bulbar muscles and respiratory muscles. Nearly all patients had detectable AChR antibodies, but no MuSK (MG patients have antibodies against the muscle‐specific kinase, MuSK) or LRP4 (MG patients have antibodies against the agrin receptor low‐density lipoprotein receptor‐related protein 4, LRP4), similar to studies about TAMG.^6^ Initial studies described that in 10%–20% of MG patients, thymoma occurred and the age range of such patients was 40–60 years.^2^, ^21^ Alvarez‐Velasco et al. have found that more frequent generalized symptoms and myasthenic crises occurred in patients with thymomatous MG, for which these patients were worse in their prognosis even after thymectomy.^6^ Besides, these patients with positive AChR IgG might also have RYR IgG or Titin IgG in serum tests, such as patient 1. On the one hand, anti‐AChR Abs cause disruption of neuromuscular junction (NMJ) components’ signaling^21^ by recognizing and attacking extracellular epitopes of the post‐synaptic membrane, which leads to AChR degradation. But the AChR Ab titer has a loose correlation with prognosis of thymoma‐associated MG.^6^ On the other hand, RYR (ryanodine receptor, a Ca^2+^ release channel of the sarcoplasmic reticulum of striated muscle) and Titin (giant filamentous muscle protein) antibodies belong to non‐AChR muscle autoantibodies and present high specificity for thymomatous MG and late onset MG (age ≥ 50 years).^22^, ^23^, ^24^, ^25^

The patients in our review presented memory impairment, confusion, and seizures most commonly in AE, which were consistent with the clinical picture of limbic encephalitis (LE).^4^ LE, a specific clinical syndrome of AE with a clinical picture consisting of memory deficit, confusion, mood changes, and seizures,^4^, ^26^ was first described in 1960^27^ and was replaced by the term AE in referring to the disease entity since the autoimmune etiology has been recognized.^28^ Although the MRI of brain seemingly failed to show consistent and specific abnormalities in AE patients, temporal lobes were highly involved, which also met the MRI criteria of LE in the previous study.^4^ Furthermore, the symptoms and the abnormalities of brain MRI are closely linked to the AE‐associated antibodies.

Antibodies which mediate AE are categorized into two types: (a) neuronal cell surface antibodies more frequently found, and (b) intracellular antibodies based on the antigens that they targeted.^29^ In our studies, patients more commonly had positive AMPAR2‐Ab or VGKC‐Ab. It is important to note that antibodies previously considered to directly bind to the voltage‐gated potassium channel (VGKC) were discovered to bind to other proteins in the VGKC complexes including LGI1, CASPR2, and Contactin‐2.^30^ Based on observations over recent years, there were 3 autoantibodies of AE associated with thymoma involving anti‐AMPAR antibody, anti‐GABA_A_ R antibody, and anti‐LGI1 antibody, which mainly targeted the limbic system in antibodies‐mediated AE.^31^ Anti‐AMPAR AE is usually described with typical limbic encephalitis and a high rate of relapse with or without tumor recurrences.^32^, ^33^ AMPARs are glutamate‐gated ion channels composed of GluR‐1, ‐2, ‐3, and ‐4 subunits. And alternative splicing and RNA editing of subunits result in different isoforms of AMPARs.^34^ Anti‐AMPAR IgG antibodies frequently targeting the GluR1/2 subunits, internalize and degrade the receptors and lead to a global decrease in AMPA receptors of the synapse.^32^ Since GluR1/2 and GluR2/3 have the highest level of expression in the hippocampus and are associated with spatial learning and memory,^34^ patients with anti‐AMPAR encephalitis had memory loss and presented clinical manifestations of LE in the review. Although anti‐GABA_B_R AE is more likely to occur with small‐cell lung cancer (SCLC), case 3 had positive GABA_B_R‐Ab.^35^ In the case presented by Alexopoulos et al.,^36^ the strong expression of GABA_B_R in the thymic epithelial cells had been proven by immunocytochemistry in the patient with thymoma‐associated anti‐GABA_B_R encephalitis. GABA_B_Rs, comprised of GB1 and GB2 subunits, are G protein‐coupled receptors for GABA (one kind of inhibitory neurotransmitter).^37^ Anti‐GABA_B_R antibodies bind the GB1 subunit and block the function of GABA_B_R.^38^ Although GB1 subunit are detected in the central nervous system (CNS) widely, the regions with the greatest level of GABA_B_R are hippocampus,^39^ which are similar to AMPARs. Being different from anti‐AMPAR encephalitis, anti‐GABA_B_R encephalitis is more often associated with seizure and status epilepticus^40^ because GABA_B_Rs mediate the inhibition of neuron unit discharges.^38^, ^39^ In addition, low titers of anti‐NMDAR antibodies can be additional antibodies of other CNS disorders possibly based on the opening of the blood‐brain barrier.^31^ The reversibility of internalization of neuron membrane receptors or function‐blocking of epitopes offers a possible explanation for the effectiveness of treatments that remove the pathological antibodies, such as intravenous immunoglobulin, plasma exchange, and immunoadsorption. The prognosis of thymoma‐associated AE is dictated by thymoma recurrences.

Patients with TAAE were mostly normal in CSF blood cell and protein examinations.^5^ With 9/18 patients in our review, mild lymphocytic pleocytosis (usually <100/mm^3^) or protein level elevation (usually <170 mg/dL) could also occur in over/approximately 50% of patients with paraneoplastic encephalitis^41^ and be explained by inflammatory changes. CSF analysis assists clinicians in making the diagnosis of immune‐mediated neurological diseases and the differential diagnosis including central nervous system infections and leptomeningeal metastases. But there was no evidence of a connection between these results of CSF and the prognosis of patients.

The presence of thymoma‐associated autoimmune complications is associated with the types of thymic pathology. For instance, autoimmune diseases are more common in patients with WHO B and AB thymoma subtypes.^42^ Histologic subtypes AB, B1, and B2 were highly associated with MG since thymoma actively exports more autoreactive T cells in these subtypes.^2^, ^43^ The expression of self‐antigens by thymic epithelial cells^1^, ^36^ and the disorder of self‐tolerance T‐cell selection through the thymus^44^ are considered as the link between tumors of the thymus and autoimmune complication. Then, the autoreactive T cells affect B cells and activate humoral immunity which further induces autoimmune diseases.^2^ As autoantibodies are produced in peripheral blood and can be independent of thymoma due to the existence of long‐lived plasma cells^21^ and long‐memory T lymphocytes,^42^, ^45^ thymoma resection does not mean remission of autoimmune diseases. MG is difficult to be completely cured because as Buckley et al found, mature and autoreactive T cells, which induce autoantibody production in MG, remained in peripheral blood for over 20 years following thymectomy.^45^ Early‐onset post‐thymoma MG (<6 months after thymectomy) is supposed to be mediated by extrathymic antibody production and late‐onset post‐thymoma MG (≥6 months) may correlate with thymoma recurrence.^46^ There was no difference in the effectiveness of thymectomy on prognosis being found between pre‐thymoma MG and post‐thymoma MG,^46^ but the long‐term clinical benefit has been proven through reductions in Quantitative Myasthenia Gravis scores and increase in patients who reached the minimal manifestation status.^47^ Patients with TAMG usually get clinical remission depending on anticholinesterase and immunosuppressants.

Compared with MG, AE is more possibly to be completely cured after thymectomy and immunotherapy without thymoma local invasion or metastasis in our studies. However, paraneoplastic AE with onconeuronal antibodies rarely responds to immunotherapy^48^ since the pathogenic mechanism mainly consists of cellular immunity mediated by cytotoxic T cells which needs to be distinguished from paraneoplastic AE with neuronal cell surface antibodies.^49^ In a retrospective cohort study conducted by Guasp and his colleagues, antibodies against intracellular antigens only occur in 30% of patients with thymoma‐associated AE.^5^ And concurrent with onconeuronal antibodies means poor outcomes among patients with anti‐AMPAR encephalitis.^5^ Previous studies also emphasized the importance of surgical removal of thymoma along with immunotherapy in TAAE.^33^, ^50^ It also reminds us of different immunopathogenesis between MG and AE. Although the mechanism was still unclear, we supposed that AE was more likely to be mediated by thymoma and thymoma recurrence rather than extrathymic antibody production. In our studies, AE arising after several years following thymectomy indicated a worse prognosis since it has more possibility to herald a tumor recurrence than MG. The long interval between thymectomy and AE onset, and the recurrence of tumor can be used to explain the poor outcome of patient 1 compared with patient 2. The research of Guasp et al also showed that 30% of TAAE had a previous diagnosis of MG and 51% of TAAE was complicated with thymoma invasion or metastasis.^5^ And MG onset often led to thymoma detection and correlated with early Masaoka stage (I and II).^3^ On the other hand, neurotropic virus infections may trigger AE through polyclonal immunoreaction,^51^, ^52^ which means antibodies are produced in polyspecific immune response against non‐causative antigens of CNS. The association between herpes virus family and neurological autoimmune disease has been described in several research, such as HSV and AE,^53^ EBV and multiple sclerosis (MS),^52^ EBV and AE.^51^ Since 3 gene sequences of EBV were detected in CSF of patient 1, the elapsed infection of EBV might also contribute to the pathogenesis of AE. However, the role of elapsed CNS infection in thymoma‐associated AE is still unknown.

The myogenic damage showed by the electromyography suggested that the patient in case 2 might have simultaneous onset of MG and inflammatory myositis (IM). The fluctuated muscle weakness with the normal level of serum muscle enzymes, the well therapeutic responses to cholinesterase inhibitor, and the positive serum AChR Abs still supported the diagnosis of MG in case 2. The diagnosis of inflammatory myopathy (IM) was unclear due to the lack of medical records about positive antibodies related to IM. It seems that MG or IM is typically present solely. But the co‐existence of the diseases is a concomitant inflammatory phenomenon related to thymoma according to the case series reported by Huang et al.^54^ And the abnormal T‐cell activation is part of the potential pathogenesis of both MG and IM.^55^ Other thymoma‐associated autoimmune diseases include thyroid dysfunction, systemic lupus erythematosus (SLE), rheumatoid arthritis (RA), alopecia areata, pure red cell aplasia (PRCA), polymyositis, and Good's syndrome, and so on.^3^, ^56^ Thyroid dysfunction was the most frequent autoimmune disorder except MG in thymoma‐associated autoimmune disease^3^ and was related to an increasing incidence of mild MG that both thyroid dysfunction and mild MG belong to mild autoimmune processes.^57^ The effect of thymectomy on different autoimmune diseases remains varied.^3^ In Bernard's series of 85 patients with thymoma, 8.2% of patients developed an autoimmune disease after thymectomy.^3^ A cohort study of 445 patients with TAMG showed that Hashimoto's thyroiditis and rheumatoid arthritis (RA) were the most common post‐thymectomy autoimmune diseases which should be further monitored.^56^

Finally, the systematical study is rare because of the lack of cases of thymoma complicated with MG and AE, and our study showed the features of these patients were similar to thymoma‐associated MG and thymoma‐associated AE. But more similar cases are needed to further summarize the clinical manifestations of these patients.

## 5 CONCLUSIONS

Our case series is limited by the small number of cases and the retrospective data, so a specific conclusion was difficult to be drawn from it. Despite the limitation, the clinical details described in this present manuscript suggested the complexity of the relationship among AE, thymoma, and MG. This article overviews the existing cases and explores the management of these patients. The long‐term follow‐up for these patients after thymectomy was also emphasized.

## AUTHOR CONTRIBUTIONS

MS: conception, study design, data collection, analysis, interpretation of results, figure design, article draft writing, article review, and editing; QL and ZW: study design, data collection, analysis, interpretation of results, figure design, article draft writing, article review, and editing; HF and HZ: project administration and supervision, article review, and editing. All authors commented upon the manuscript draft and approved the final manuscript. The work reported in the article has been performed by the author unless clearly specified in the text.

## CONFLICT OF INTEREST STATEMENT

The authors have no conflicts of interest to disclose.

## INFORMED CONSENT

Informed consent was obtained from all patients or their families included in the case series.

## Supporting information


Appendix S1

Figure S2

## Data Availability

The data that support the findings of this study are available on request from the corresponding author. The data are not publicly available due to privacy or ethical restrictions.
