# Supplementary material for: Thymoma‐associated autoimmune encephalitis with myasthenia gravis: Case series and literature review
Source: CNS Neurosci Ther. 2024 Feb 7;30(2):e14568. doi: 10.1111/cns.14568 (PMC10850820; doi:10.1111/cns.14568)
Supplement: Supplementary file 1 — Appendix S1 [file CNS-30-e14568-s001.docx]

**Table S1. Clinical characteristics of 18 patients with TAAE and TAMG**

| Feature No. (% of reported) | Total | Good Outcome | Bad Outcome |
| --- | --- | --- | --- |
| **Sex** | 18 | 12 | 6 |
| Male | 7 (38.89%) | 4 | 3 |
| Female | 11 (61.11%) | 8 | 3 |
| **Age (years)^*^** | 18 | 12 | 6 |
| At thymoma diagnosis | 44.5 [19, 69] | 41[19, 69] | 46.5[41, 49] |
| **MR abnormalities** | 11 (61.11%) | 8 | 3 |
| **Chronological sequence** | 18 | 12 | 3 |
| Simultaneity | 6 (33.33%) | 5 | 2 |
| MG firsrt | 10 (55.56%) | 6 | 4 |
| AE first | 2 (11.11%) | 2 | 0 |
| **Thymoma histopathology** | 18 | 12 | 6 |
| A | 0 | 0 | 0 |
| AB | 2 (11.11%) | 2 | 0 |
| B1 | 2 (11.11%) | 2 | 0 |
| B2 | 5 (27.78%) | 3 | 2 |
| B3 | 0 | 0 | 0 |
| C | 0 | 0 | 0 |
| Sclerosing thymoma | 1(5.56%) | 0 | 1 |
| Lymphoepithelial thymoma | 2(11.11%) | 2 | 0 |
| Noninvasive spindle cell thymoma | 1(5.56%) | 0 | 1 |
| NA | 5 (44.44%) | 0 | 2 |
| **Thymoma recurrence** | 18 | 12 | 6 |
| Yes | 5 (27.78%) | 1 | 4 |
| No | 13 (72.22%) | 13 | 2 |
| **Remission** | 18 | 12 | 6 |
| Yes | 12 (66.67%) | 12 | 0 |
| No | 6 (33.33%) | 0 | 6 |
| **Clinical manifestation** | 18 | 12 | 6 |
| Seizure | 7 (38.89%) | 5 | 2 |
| Confusion | 10 (55.56%) | 8 | 2 |
| Memory deterioration | 13 (72.22%) | 11 | 2 |
| **Weakness of muscle groups** | 18 | 12 | 6 |
| Ocular muscles | 15 (83.33%) | 11 | 4 |
| Facial muscles | 1 (5.56%) | 1 | 0 |
| Bulbar muscles | 9 (50%) | 7 | 2 |
| Neck muscles | 2 (11.11%) | 1 | 1 |
| Respiratory muscles | 5 (27.78%) | 2 | 3 |
| Limb muscles | 4 (22.22%) | 3 | 1 |

Data are expressed as number and percentage, n (%); or * median [1st; 4^th^ quartile].

Abbreviation: MG = myasthenia gravis; AE = autoimmune encephalitis; MRI = Magnetic resonance imaging; Good Outcome = patients get clinical remission after treatments; Poor Outcome = patients did not get clinical remission after treatments.

**Table S2. Cerebrospinal fluid analysis of 18 patients with TAAE and TAMG**

| **Reference** | **CSF leucocyte level** | **CSF protein level** | **paraneoplastic antibodies (Hu,**  **Yo, Ri, Ma1, Ma2, CV2/CRMP5** |
| --- | --- | --- | --- |
|  | **(****cells/mm ^3^)** | **(****mg/dL)** |  |
| Our case 1 (2021) | 41 | Normal^#^ | Negative |
| Our case 2 (2021) | Normal* | Normal | Negative |
| Our case 3 (2020) | Normal | 70 | Negative |
| Monstad, et al(2009)(10) | 10 | Normal | CRMP5 |
| Khella, et a(2007)l(11) | 43 | Normal | Negative |
| Aysal, et al(2013)(12) | Normal | Normal | NA |
| Miyazaki, et al(2012)(13) | Normal | 60 | NA |
| Hor, et al  (2018)(14) | Normal | Normal | NA |
| Luo, et al  (2019)(15) | Normal | Normal | NA |
| Liu, et al  (2018)(16) | NA | NA | NA |
| Shaulov, et al  (2012)(9) | Normal | Normal | Negative |
| Li, et al  (2015)(17) | Normal | Normal | Negative |
| Hammoud, et al (2009)(18) | 70 | 101 | Negative |
| Kodama, et al(1991)(8) | Mild lymphocytic pleocytosis | Normal | NA |
| Evoli, et al(1999)(19) | 25 | Normal | Negative |
| Buckey, et al(2001)(20) | Normal | Normal | NA |
| Vernnino, et al(2002)(21) | 10 | Normal | NA |
| Vernnino, et al(2002)(21) | NA | NA | NA |

Abbreviation: CSF = cerebrospinal fluid; Normal* = ≤ 5 cells/mm 3; Normal^#^= ≥ 50 mg/dL; CRMP5 = Collapsin response mediator protein 5; NA = data not available.


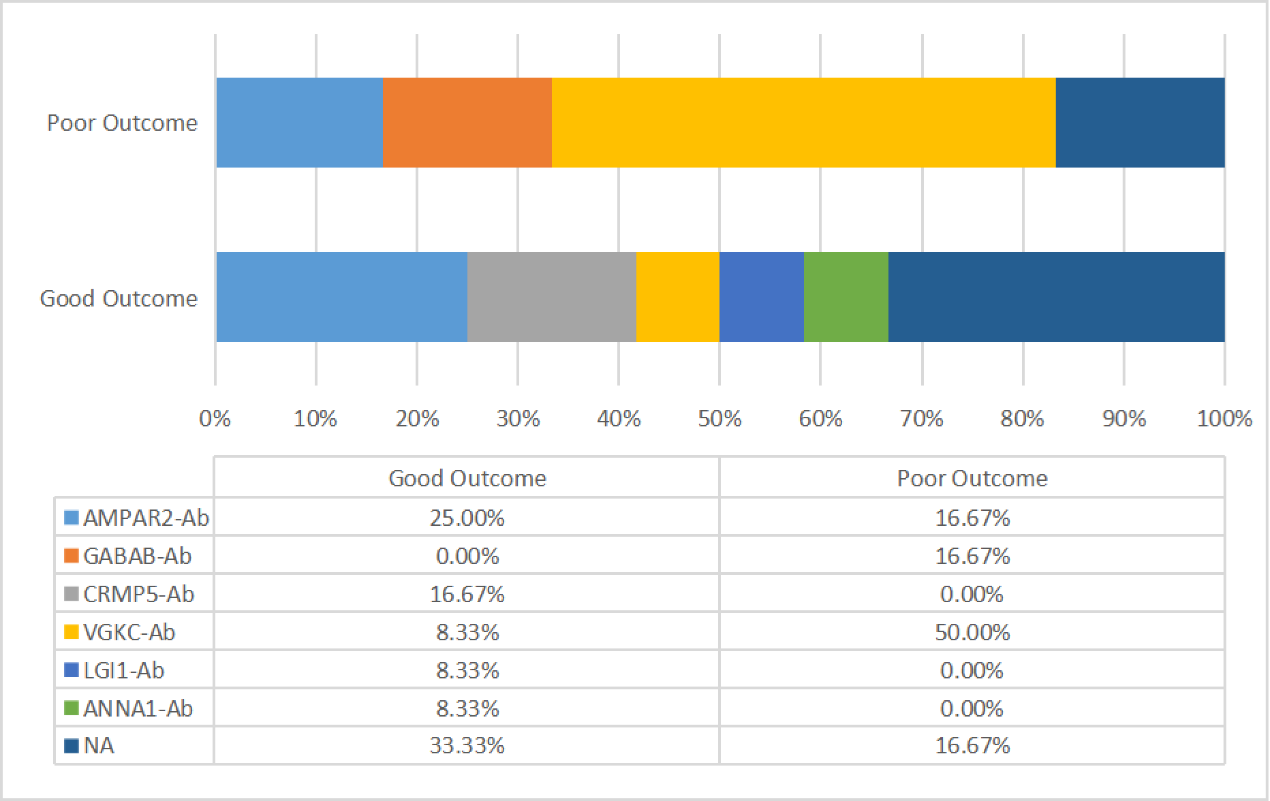


**Figure S1. AE-associated antibodies types in different groups**

Figure S1 illustrated that the types of AE-associated antibodies included 3/6 patients with VGKC-Ab in patients with poor outcome higher than patients with good outcome (1/12), 1/6 AMPAR2-Ab (vs 3/12) and 1/6 GABABR-Ab (vs 0/12). One patient with good outcome who had both ANNA1-Ab and CPMP5-Ab was divided into CRMP5-Ab type.

Abbreviation: AMPAR2 = Amino-3-hydroxy-5-methyl-4-isoxazolepropionic acid receptor 2; NMDAR = anti-N-methyl-D-aspartate receptor antibody; CRMP5 = Collapsin response mediator protein 5; O = ocular muscles; LGI1 = leucine-rich, glioma inactivated 1; GABA_B_R = gamma-aminobutyric acid receptor B; ANNA1-Ab (or “anti-Hu”) = the type-1 antineuronal nuclear antibody; NA = data not available.
