# Supplementary material for: Thymoma‐associated autoimmune encephalitis with myasthenia gravis: Case series and literature review
Source: CNS Neurosci Ther. 2024 Feb 7;30(2):e14568. doi: 10.1111/cns.14568 (PMC10850820; doi:10.1111/cns.14568)
Supplement: Supplementary file 2 — Figure S2 [file CNS-30-e14568-s002.pdf]

T2-FLAIR images of brain MRI of Case 1 after AE onset

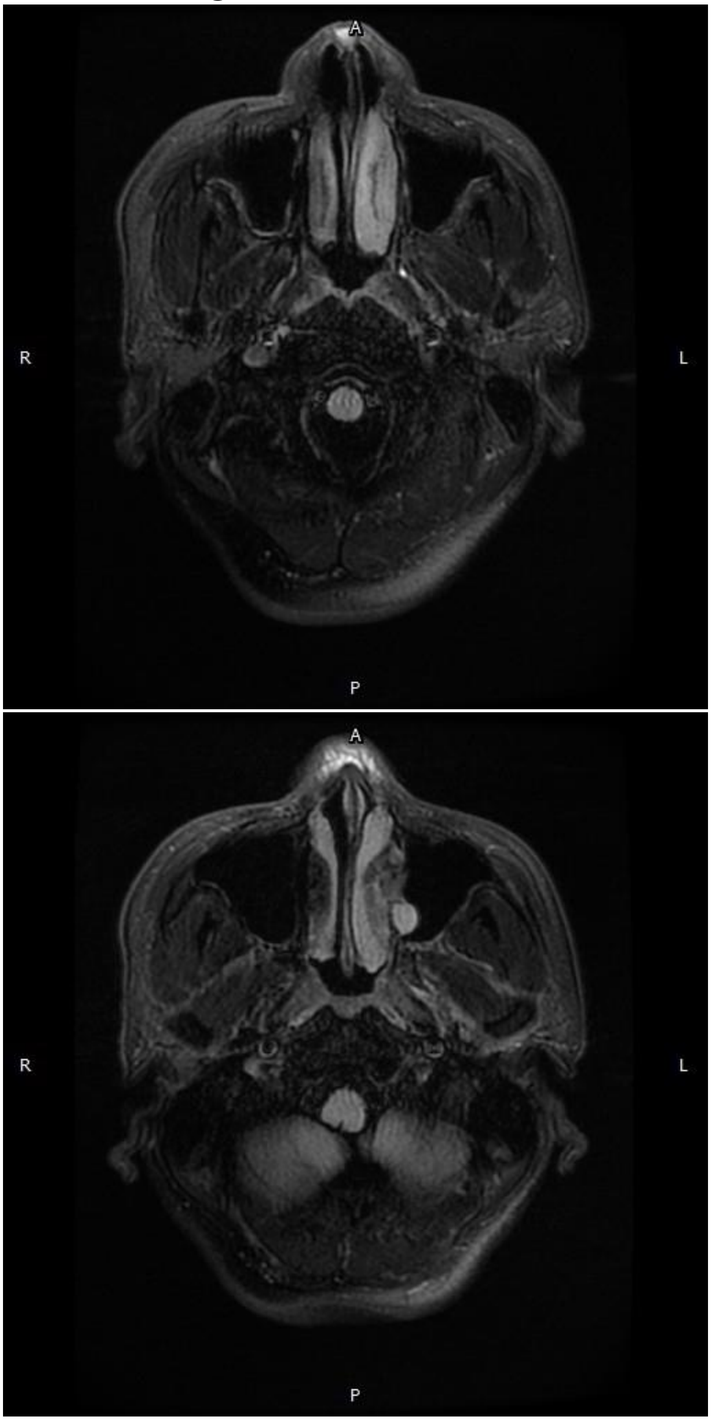

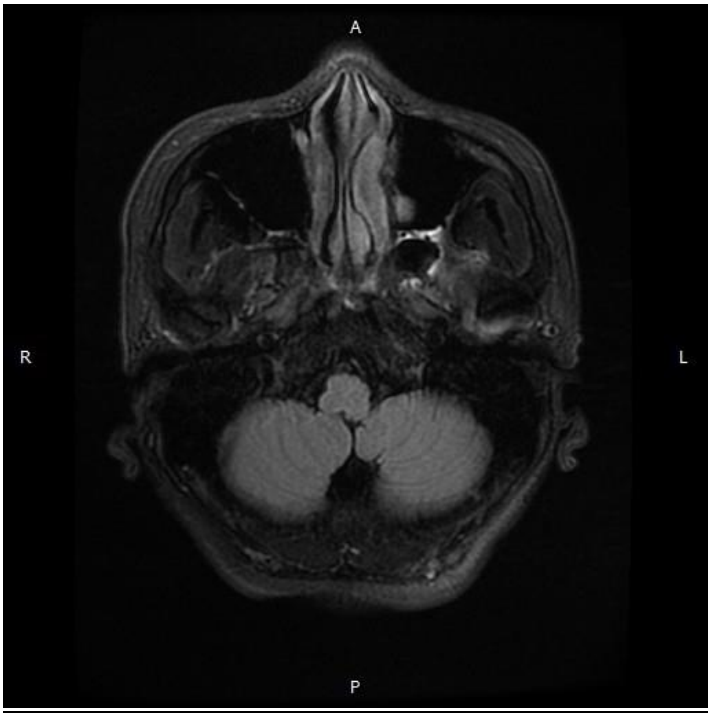
A
R
L
P

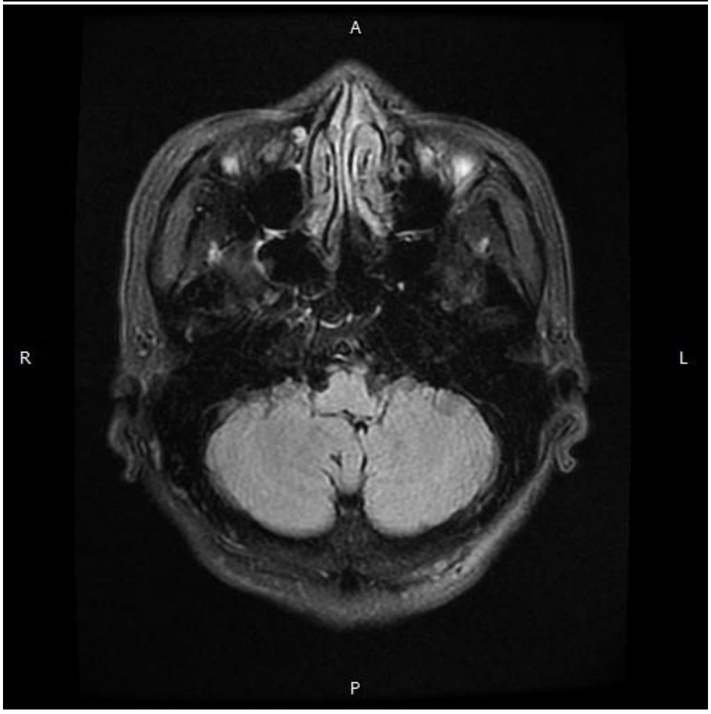
A
R
L
P

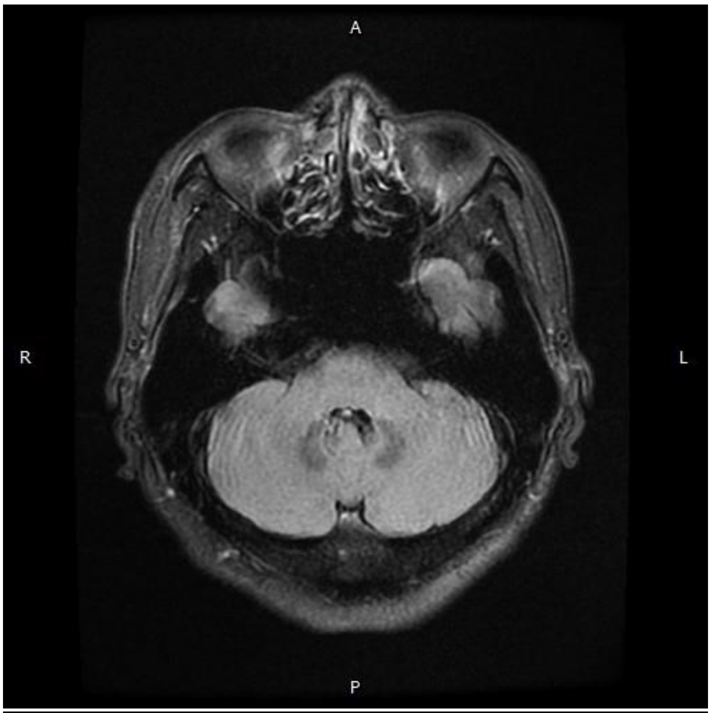
A
R
L
P

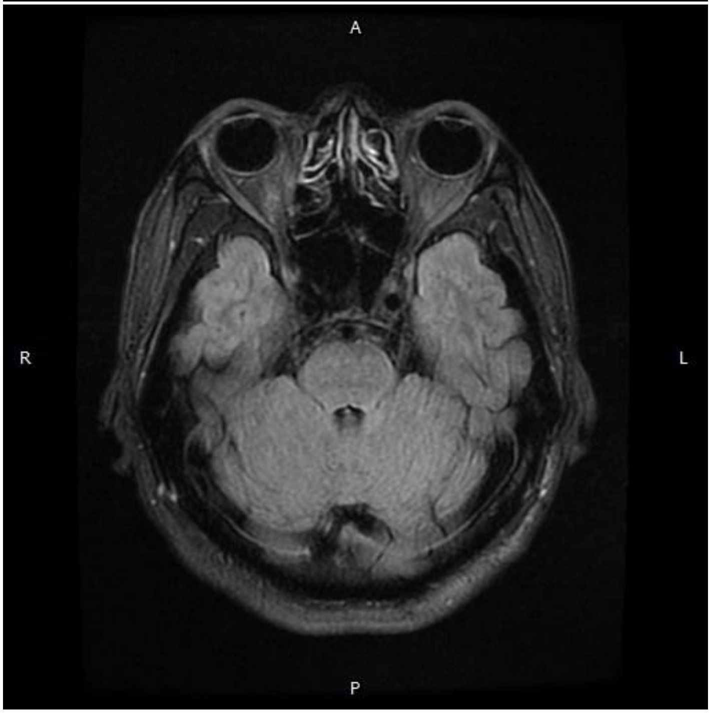
A
R
L
P

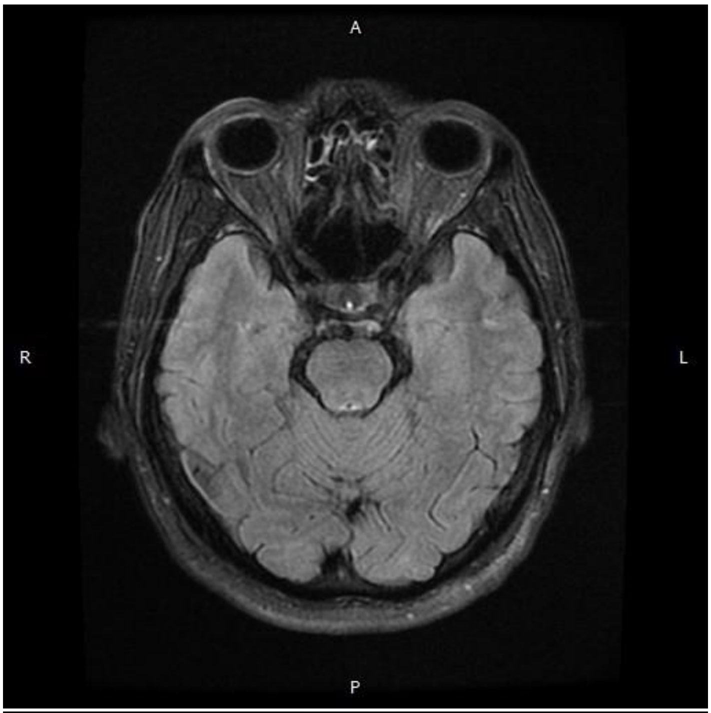
A
R
L
P

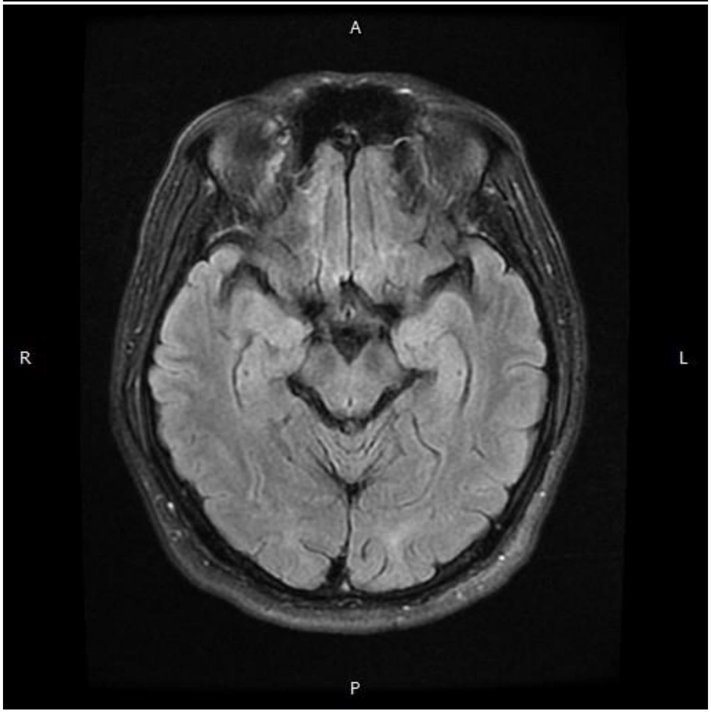
A
R
L
P

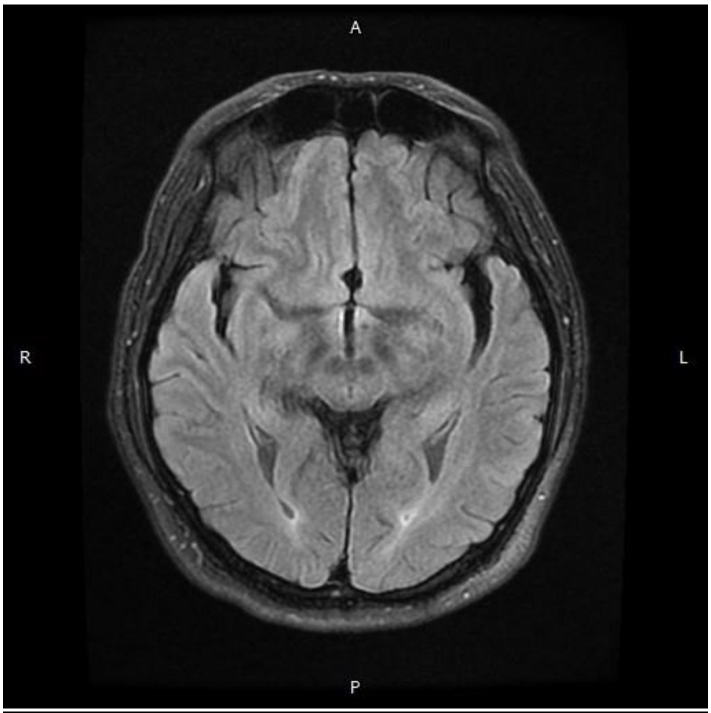
A
R
L
P

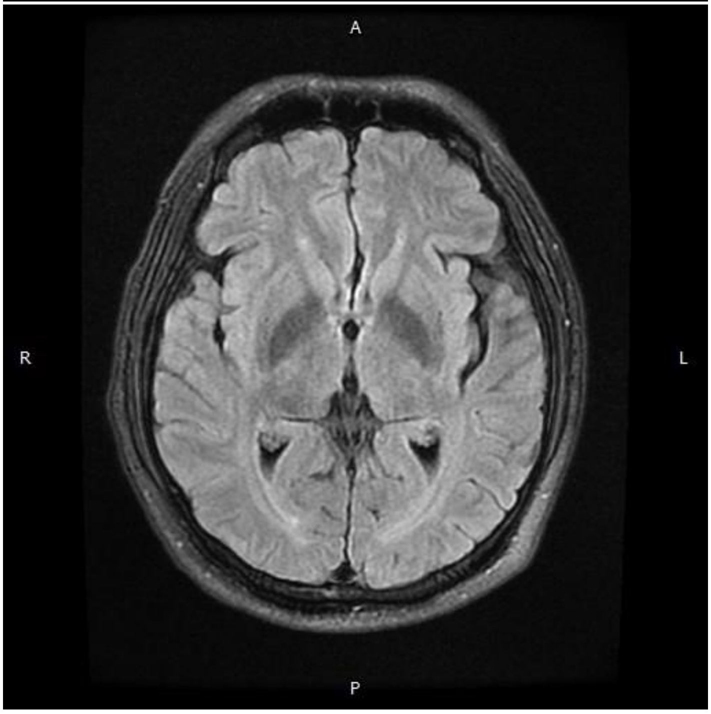
A
R
L
P

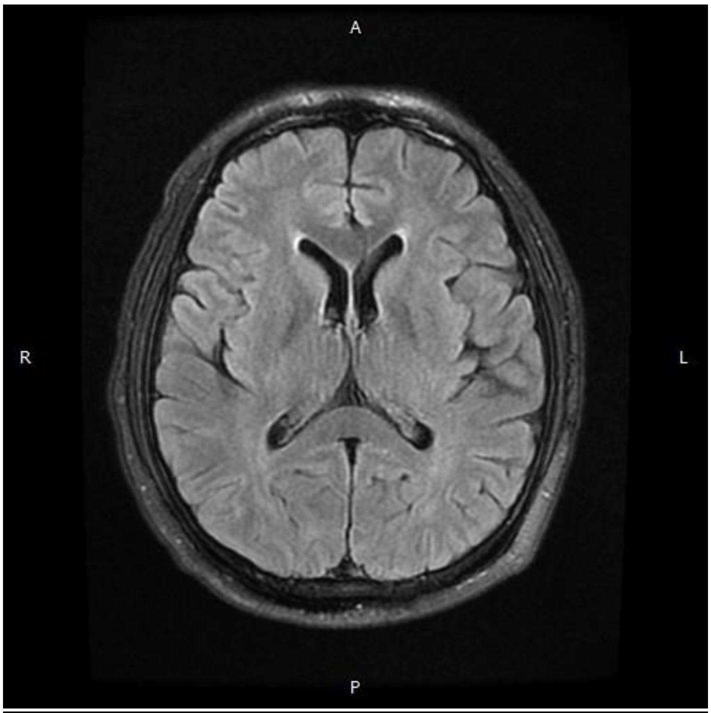
A
R
L
P

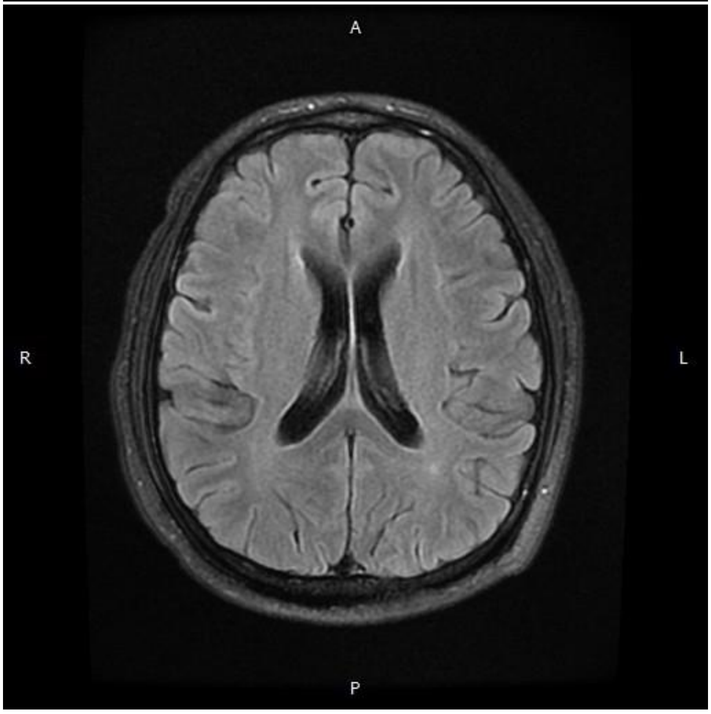
A
R
L
P

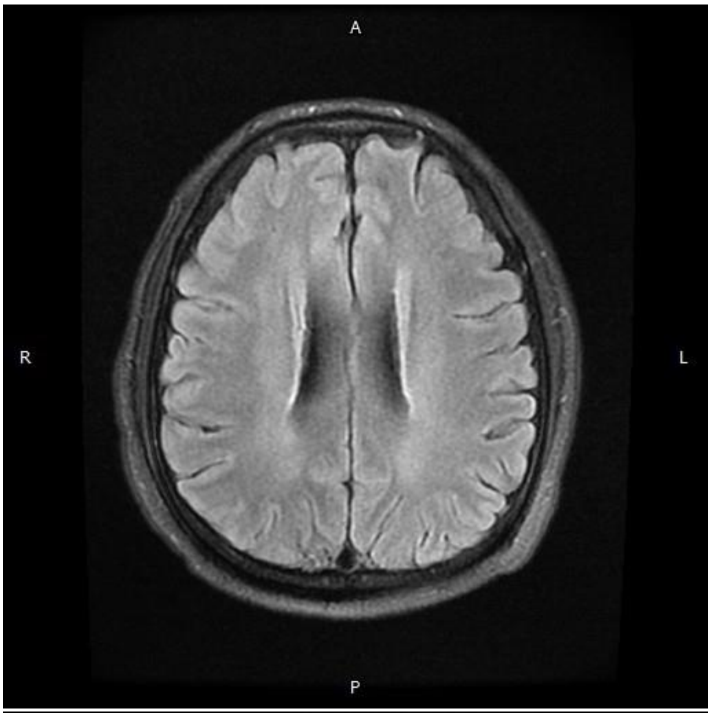
A
R
L
P
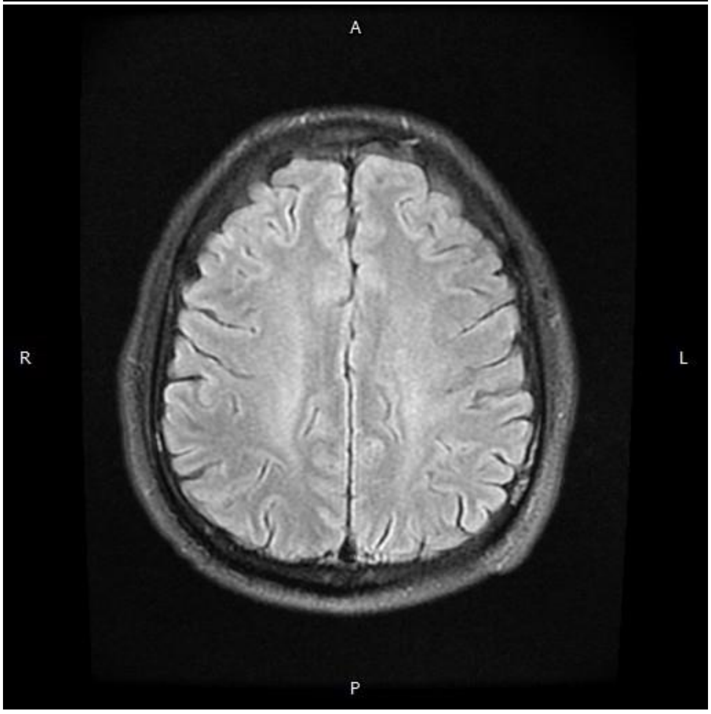
A
R
L
P

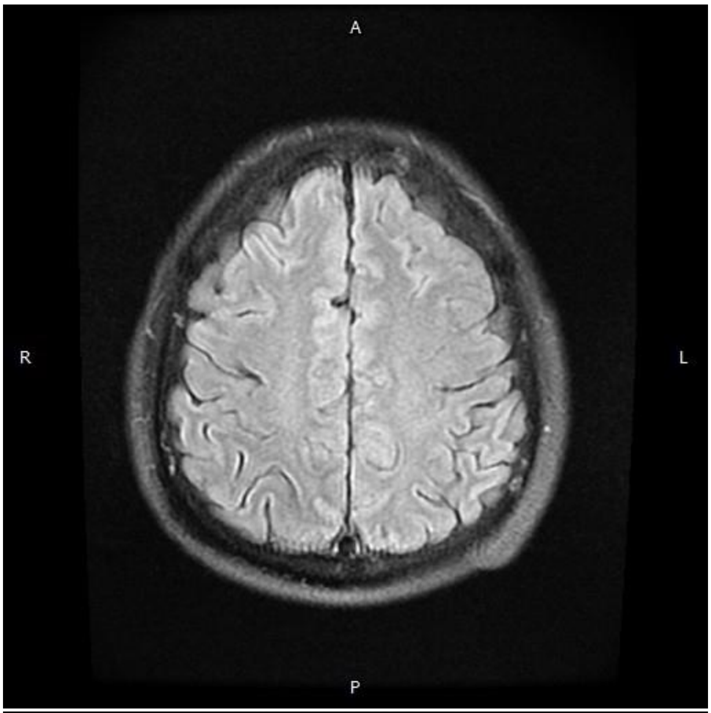
A
R
L
P

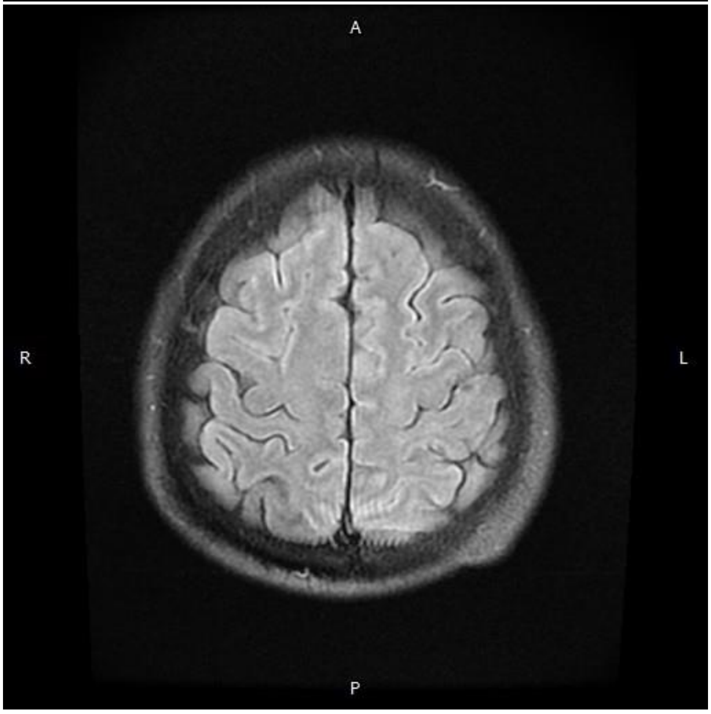
A
R
L
P

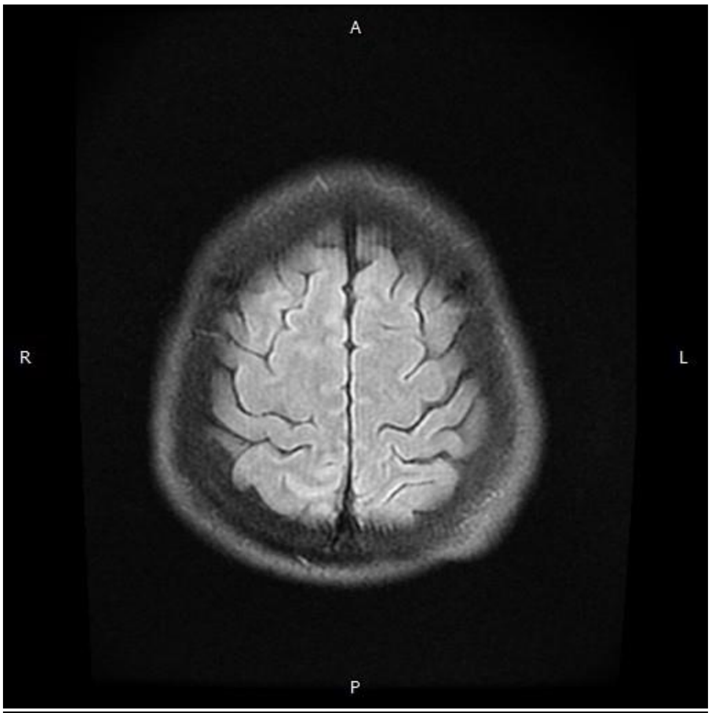
A
R
L
P

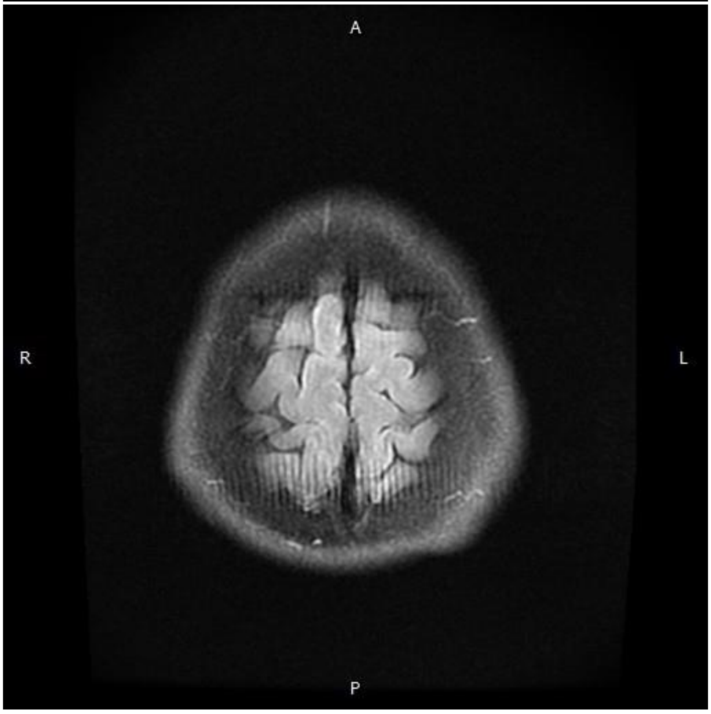
A
R
L
P

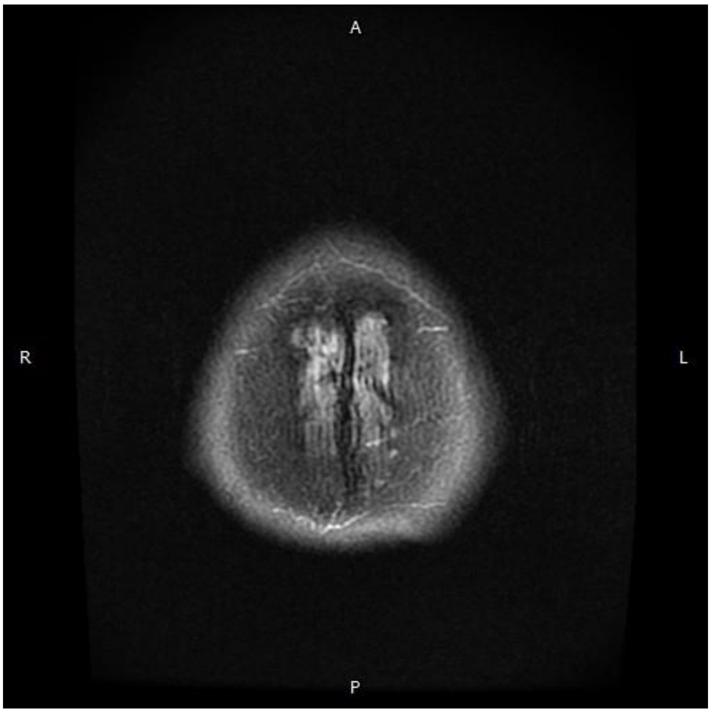
A
R
L
P

T2-FLAIR images of brain MRI of Case 2 after AE onset

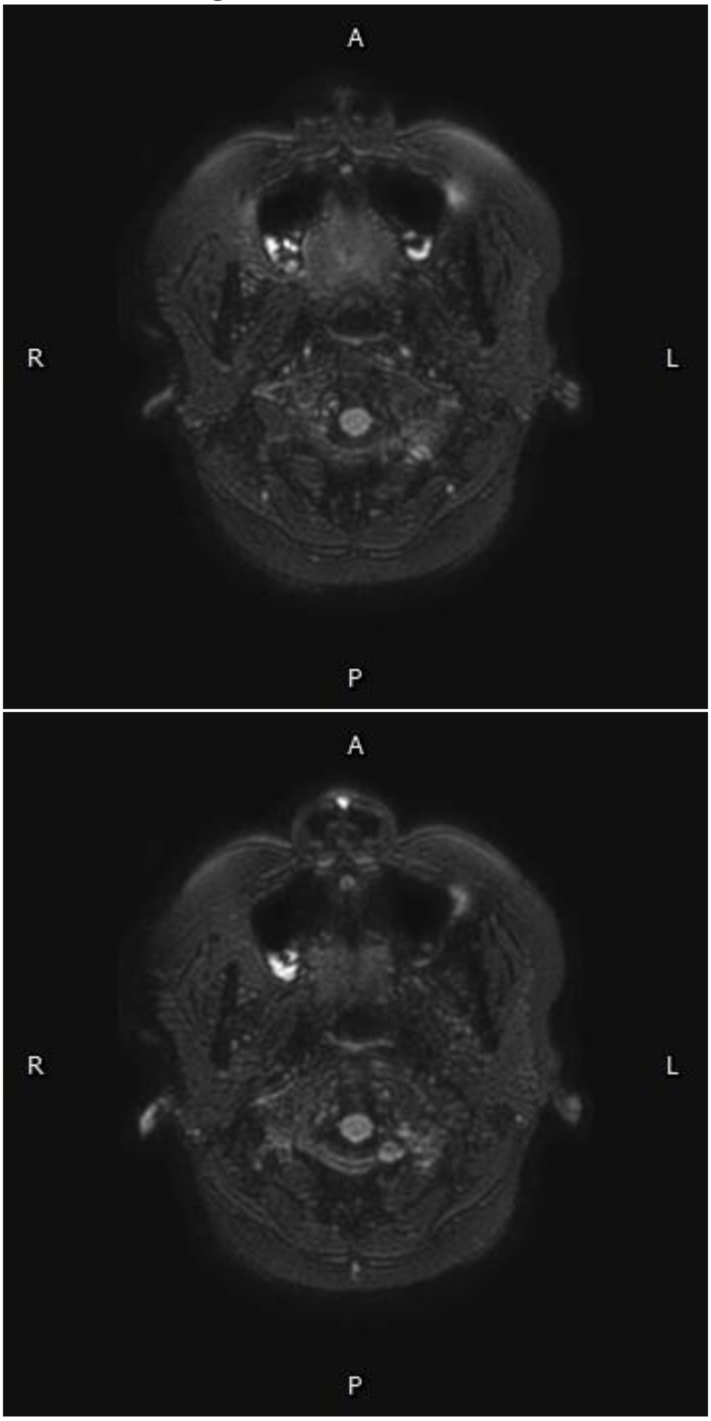

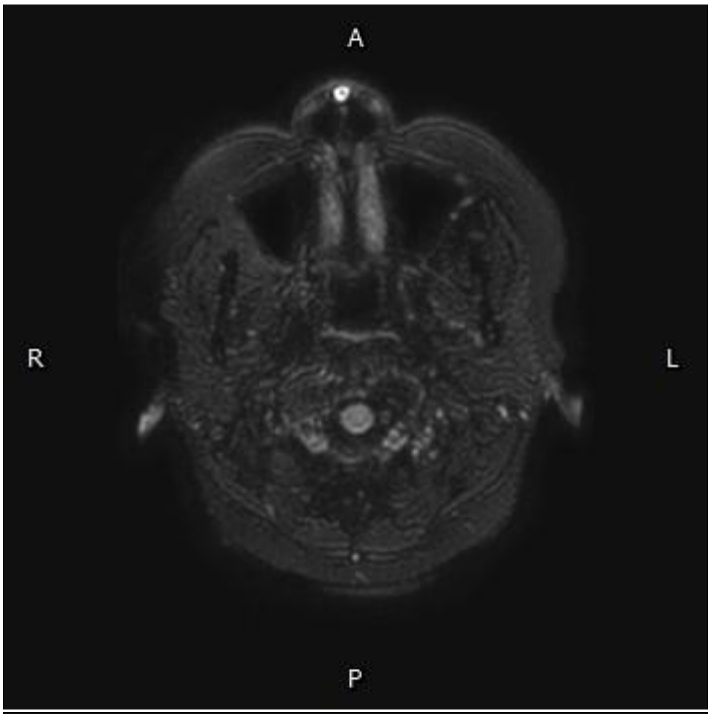
A
R
L
P

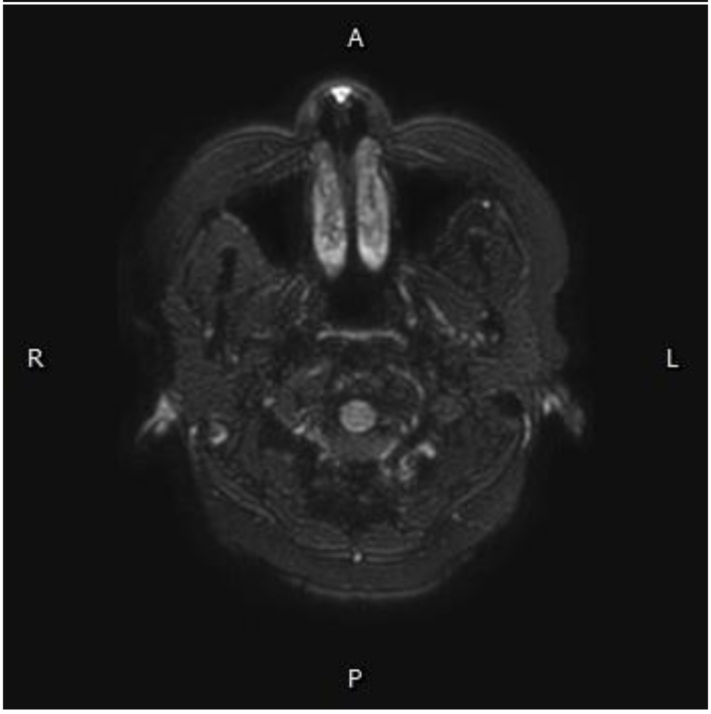
A
R
L
P

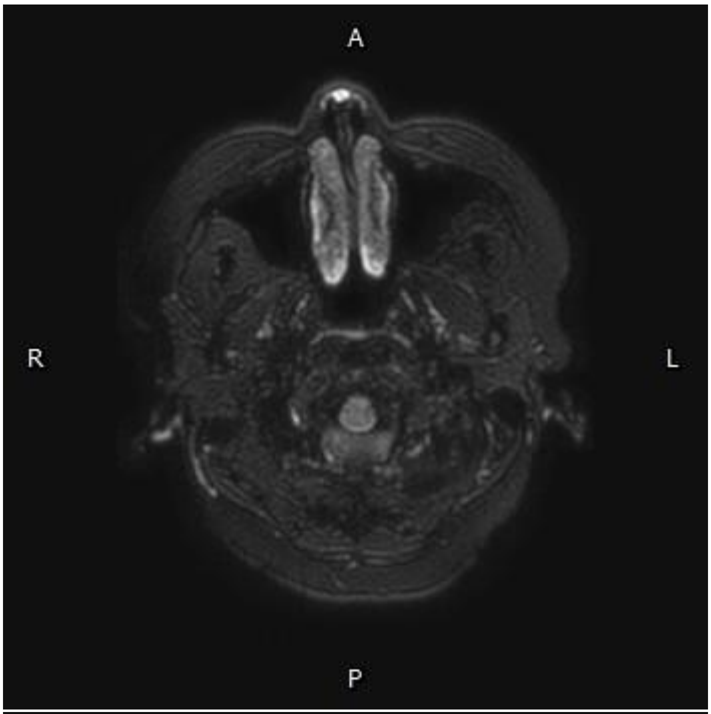
A
R
L
P
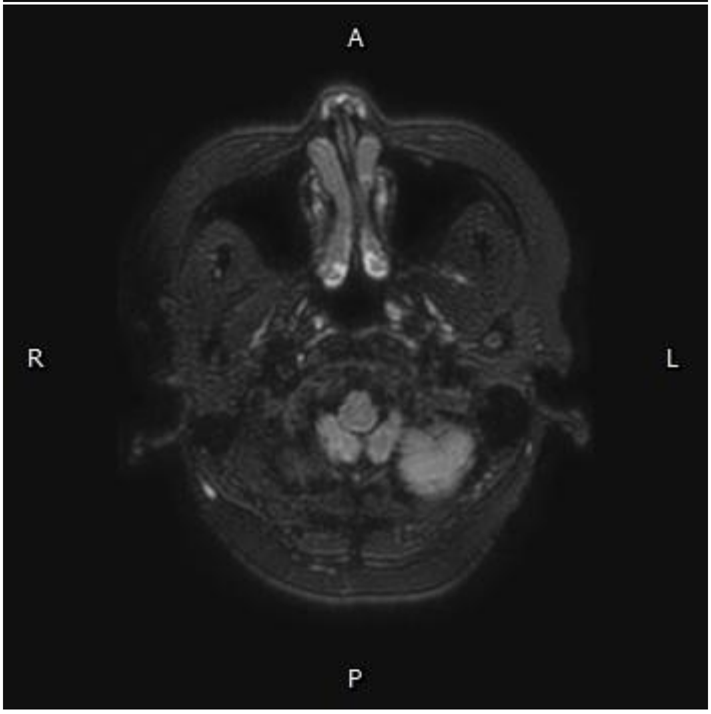
A
R
L
P

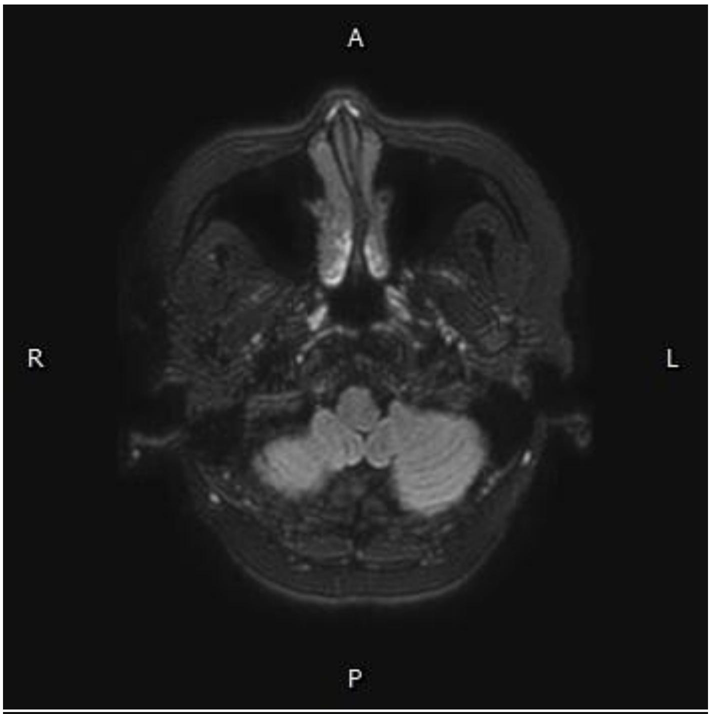
A
R
L
P
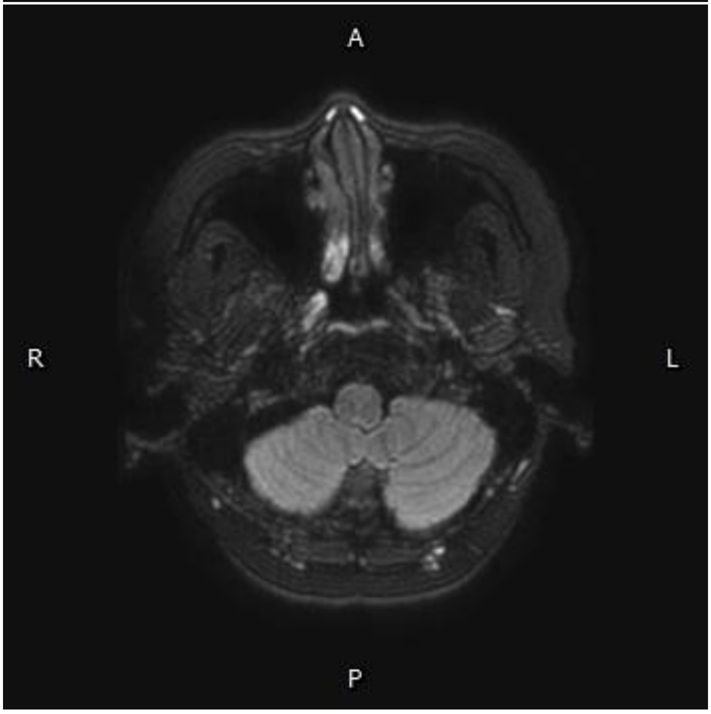
A
R
L
P

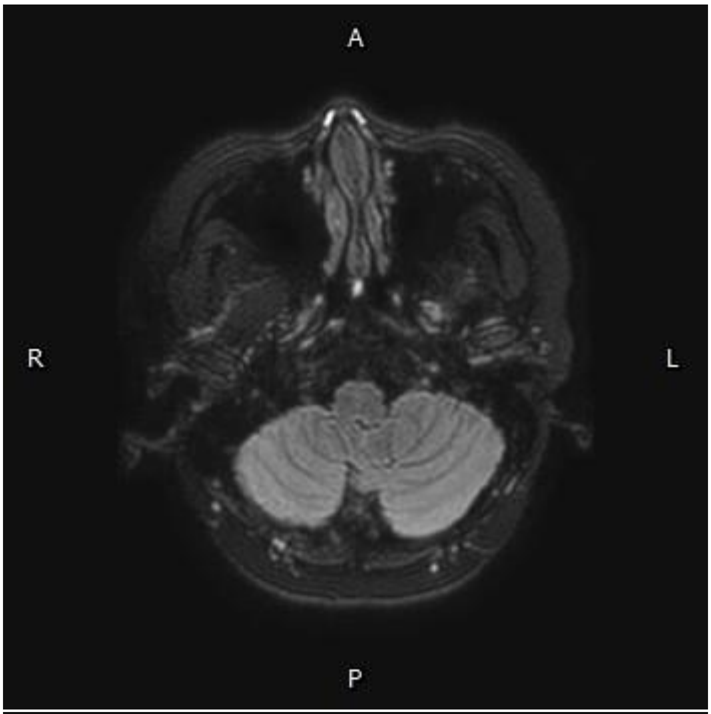
A
R
L
P

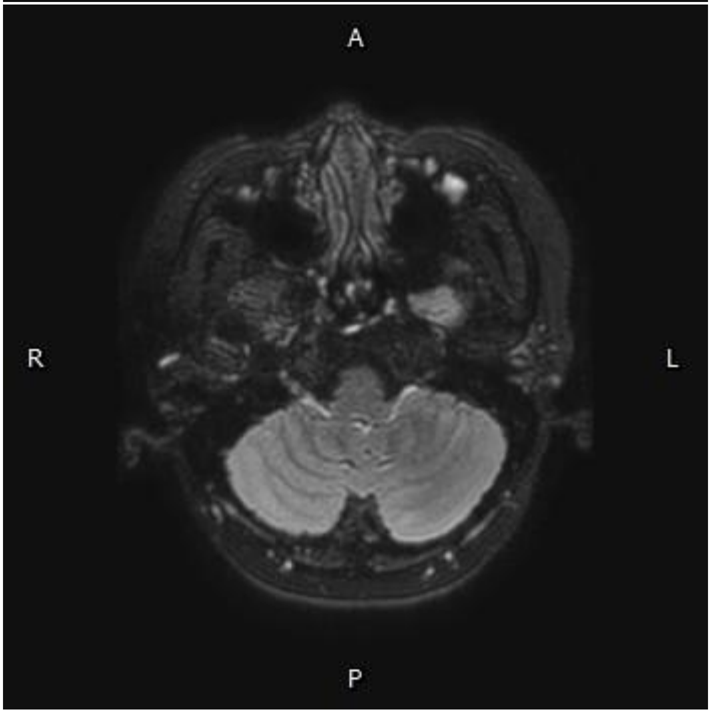
A
R
L
P

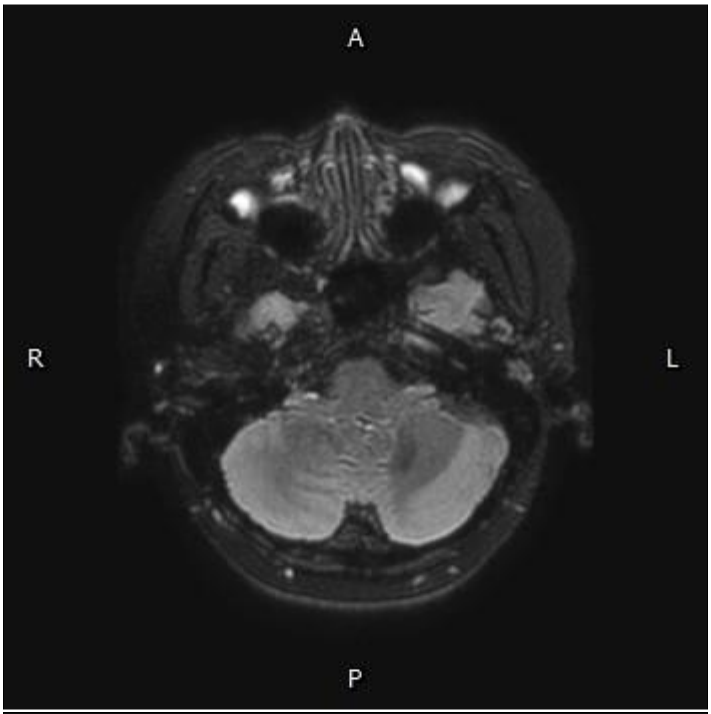
A
R
L
P

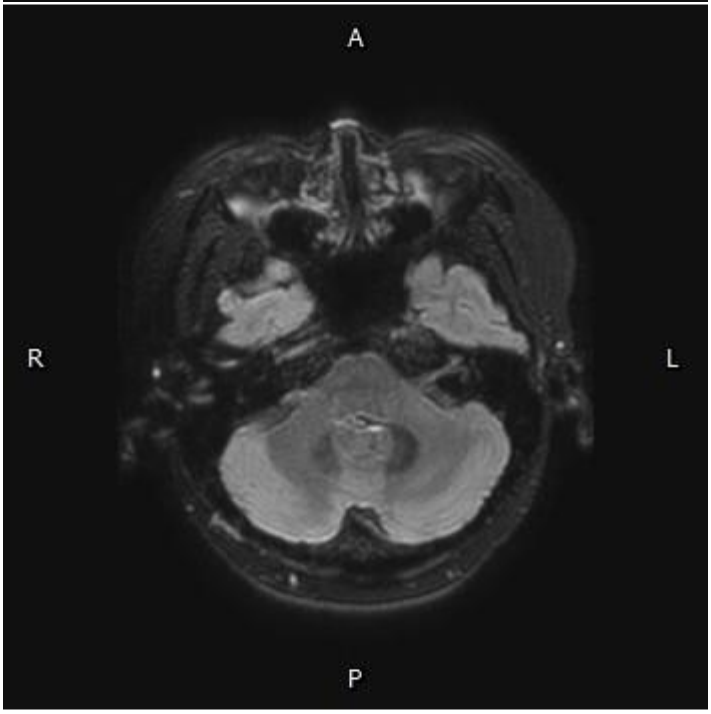
A
R
L
P

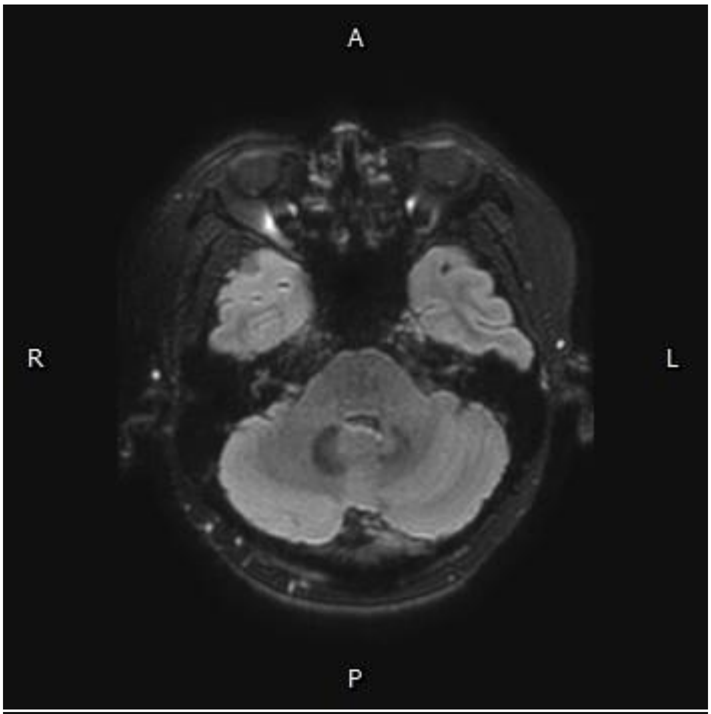
A
R
L
P
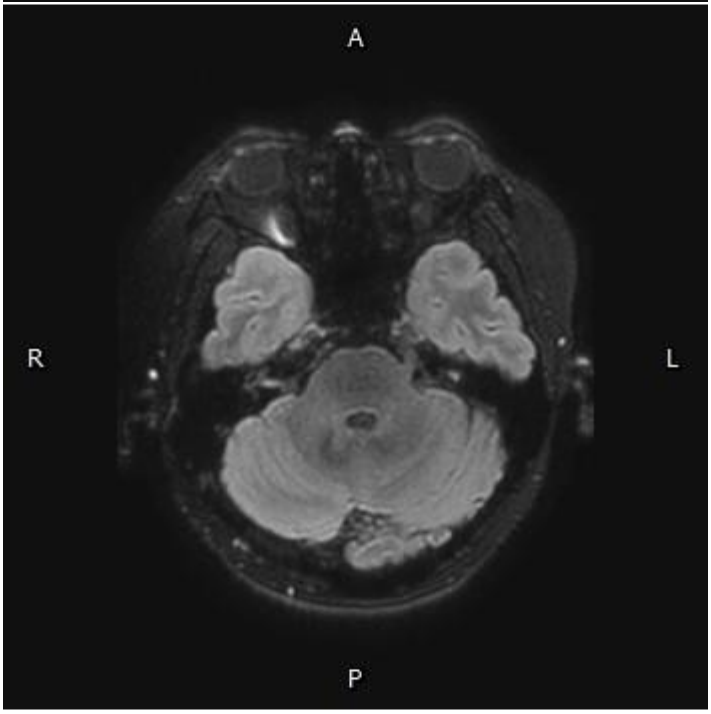
A
R
L
P

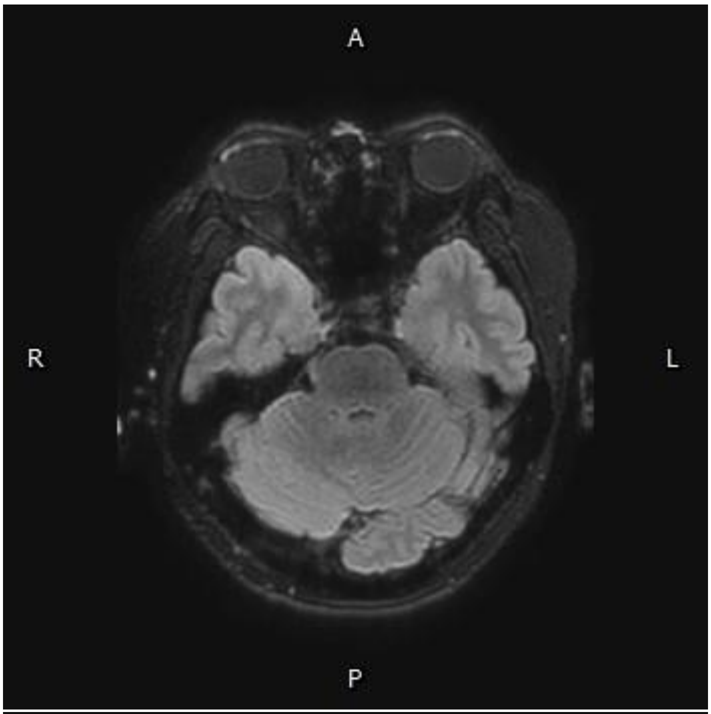
A
R
L
P
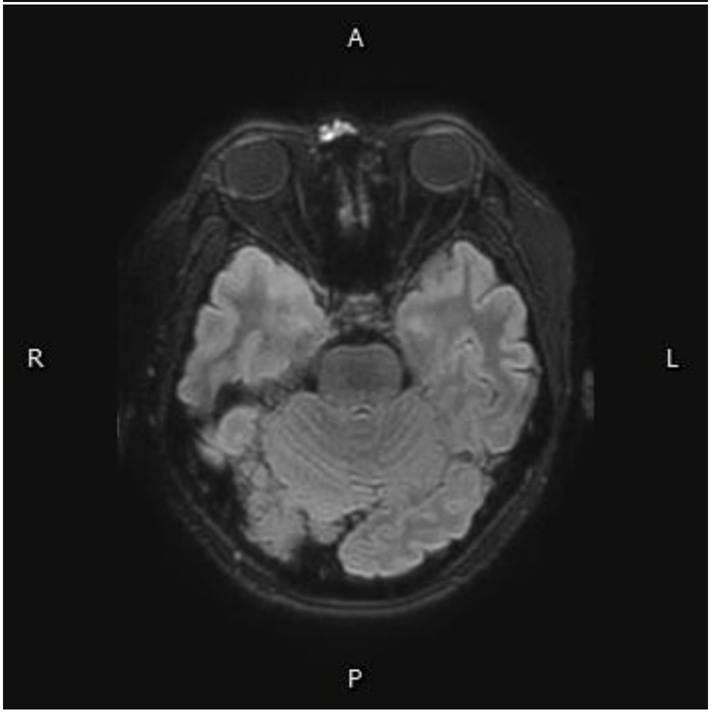
A
R
L
P

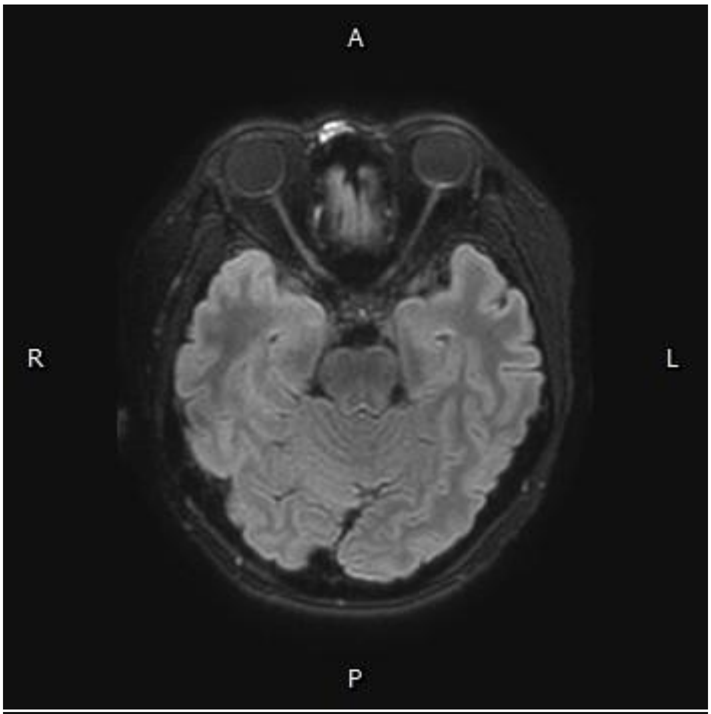
A
R
L
P

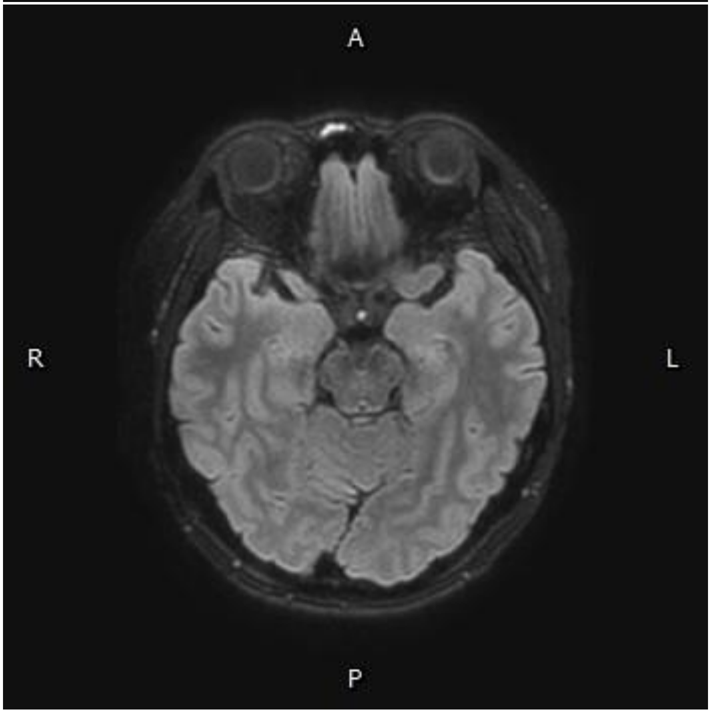
A
R
L
P

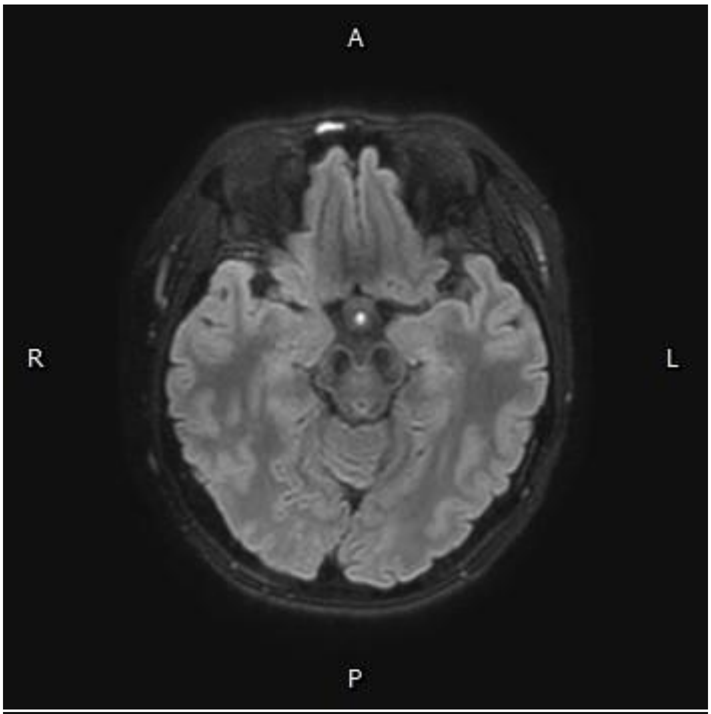
A
R
L
P

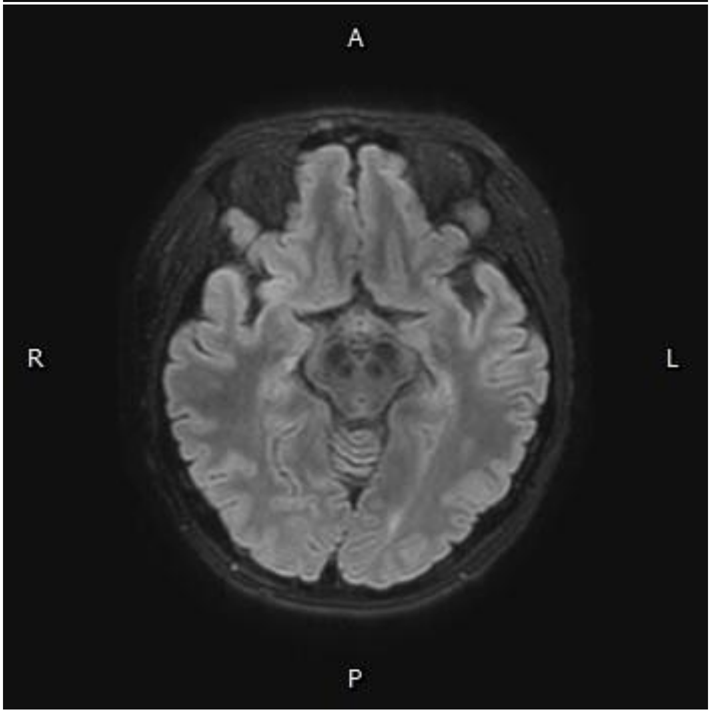
A
R
L
P

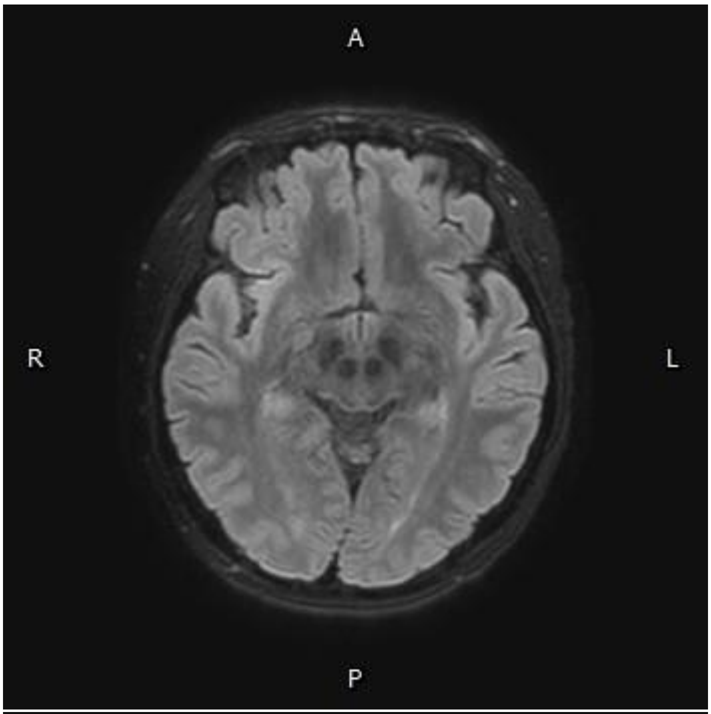
A
R
L
P

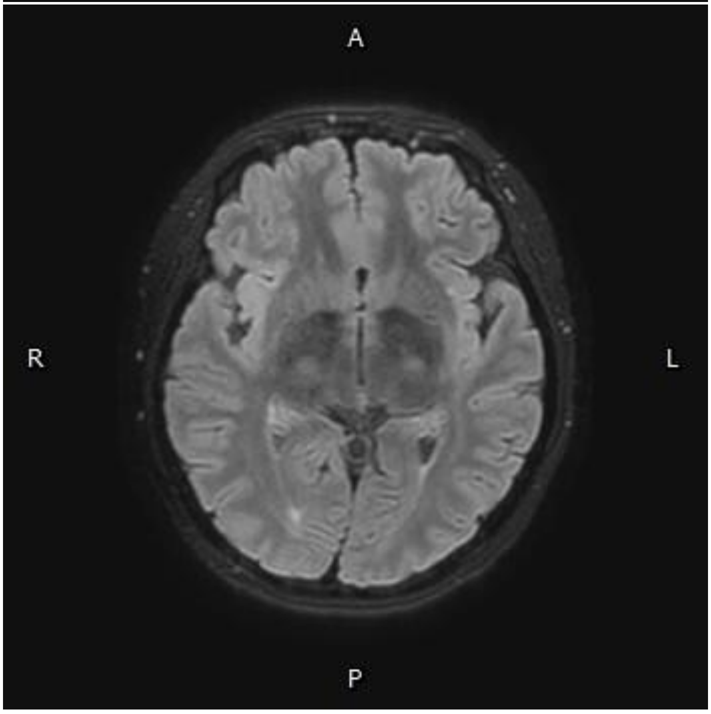
A
R
L
P

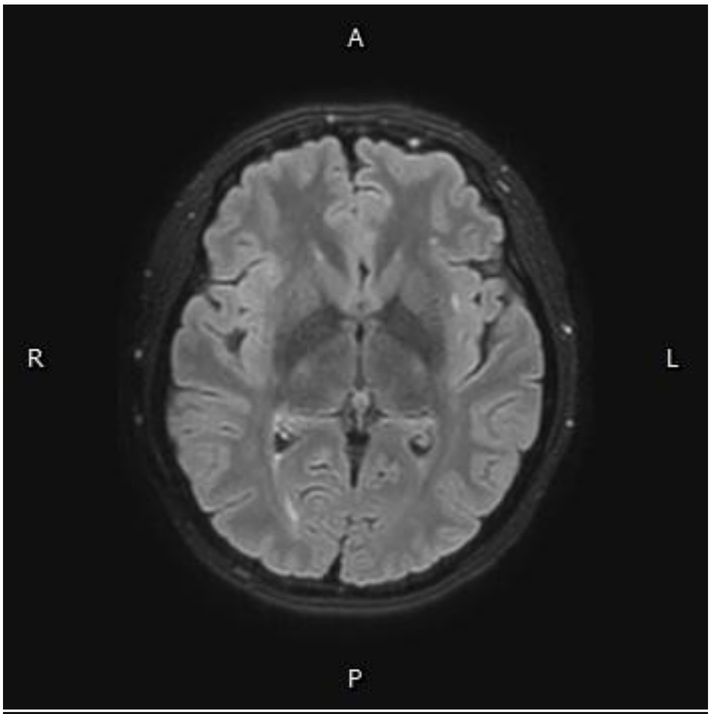
A
R
L
P

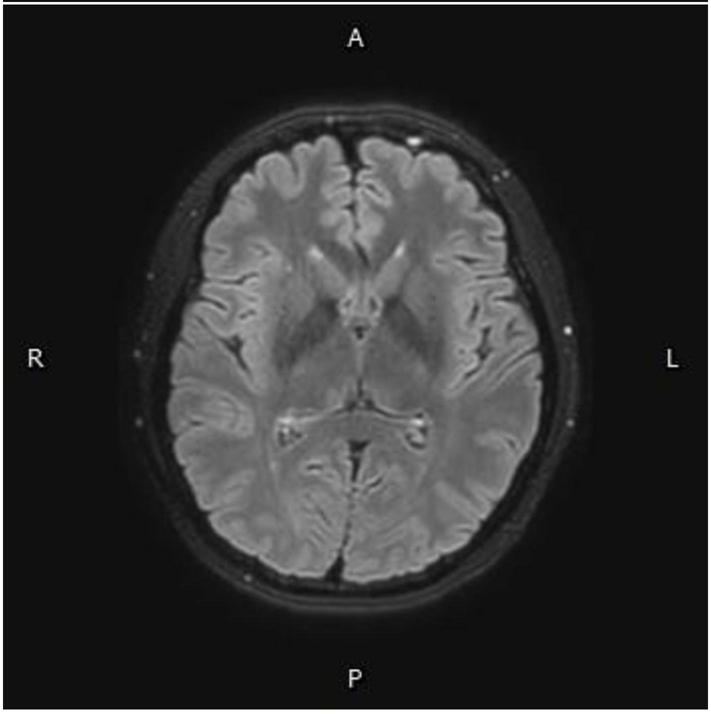
A
R
L
P

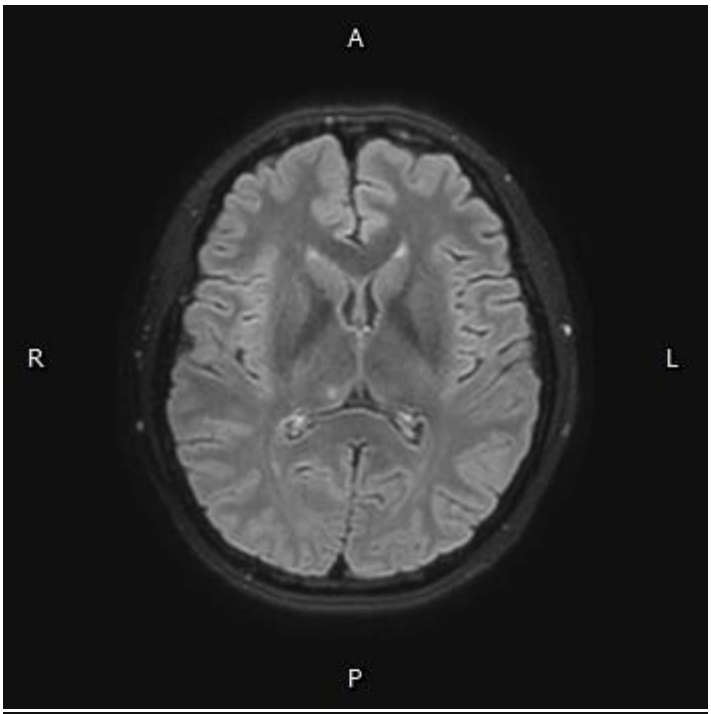
A
R
L
P

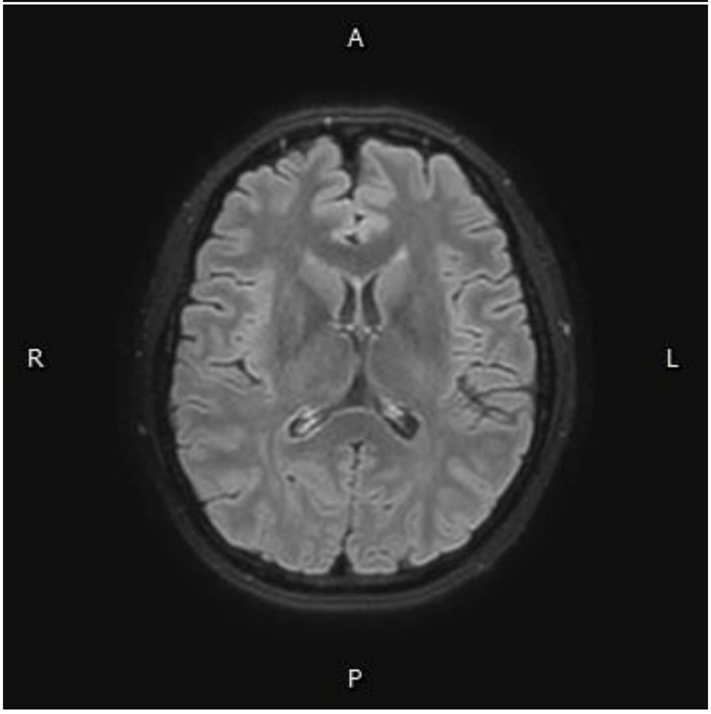
A
R
L
P

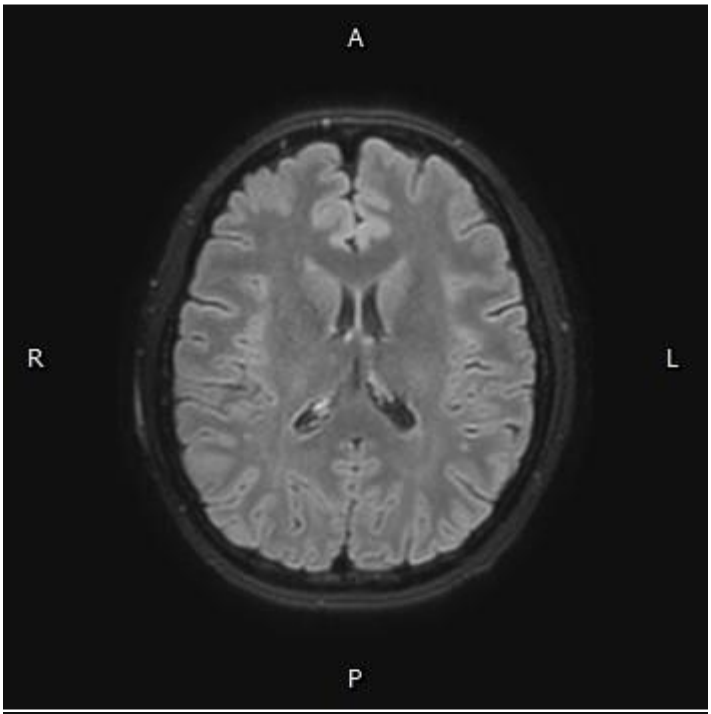
A
R
L
P

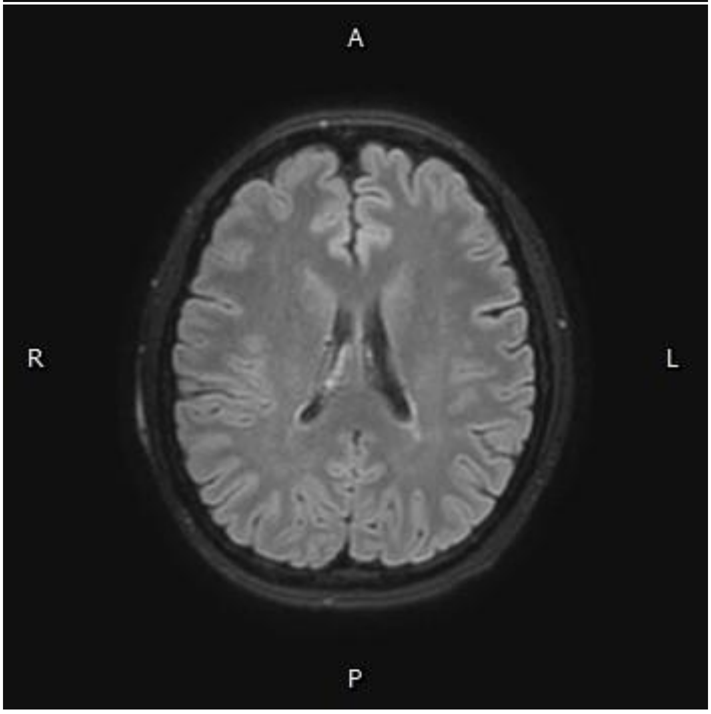
A
R
L
P

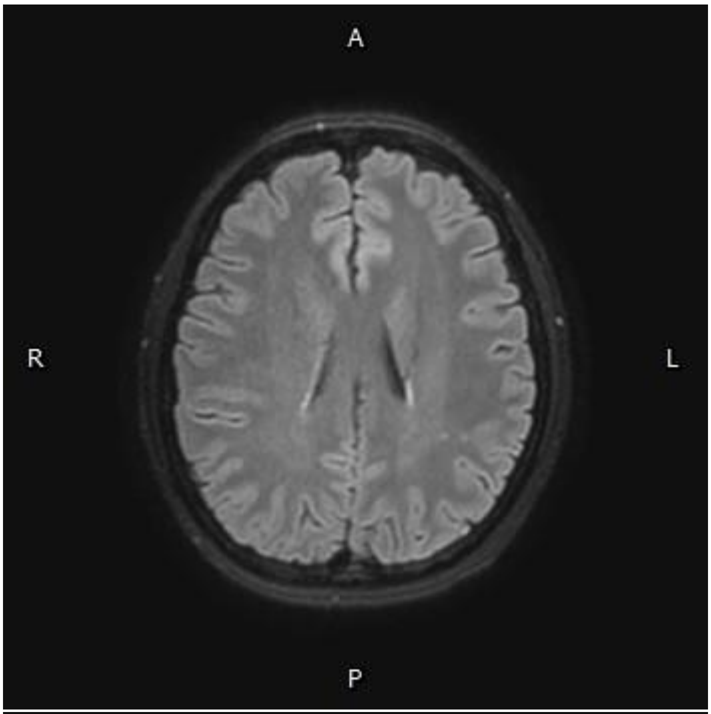
A
R
L
P

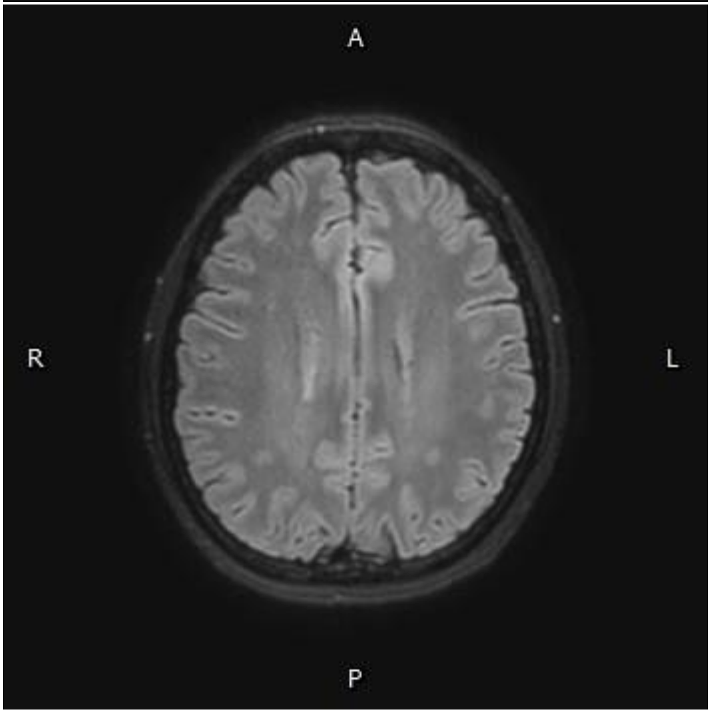
A
R
L
P

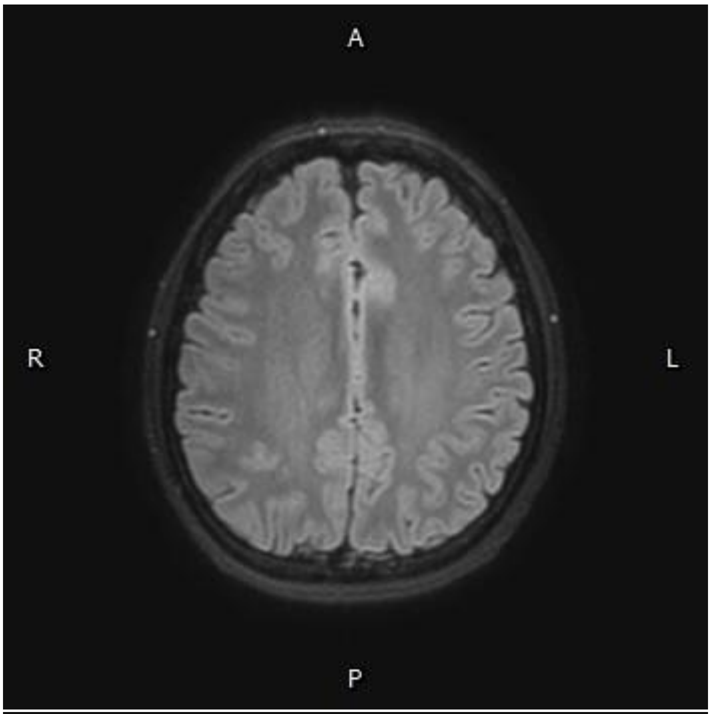
A
R
L
P

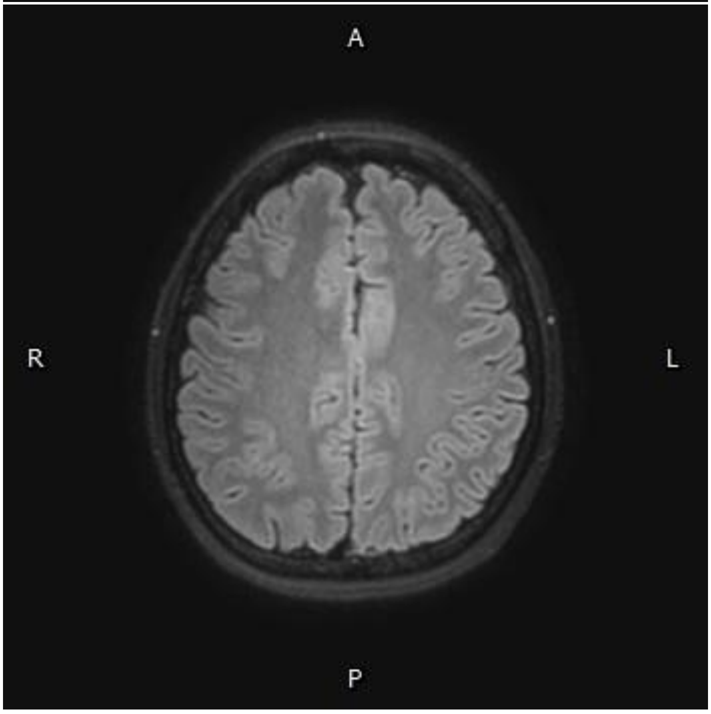
A
R
L
P

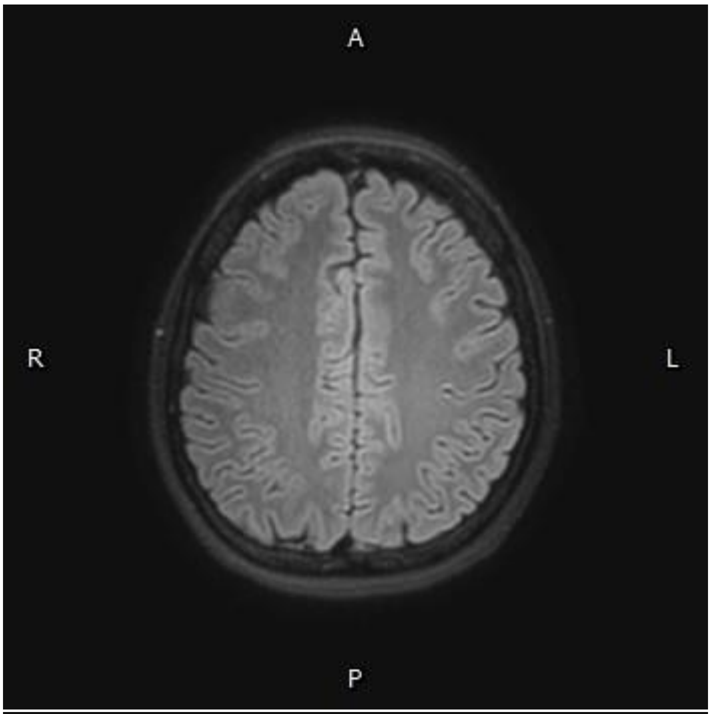
A
R
L
P
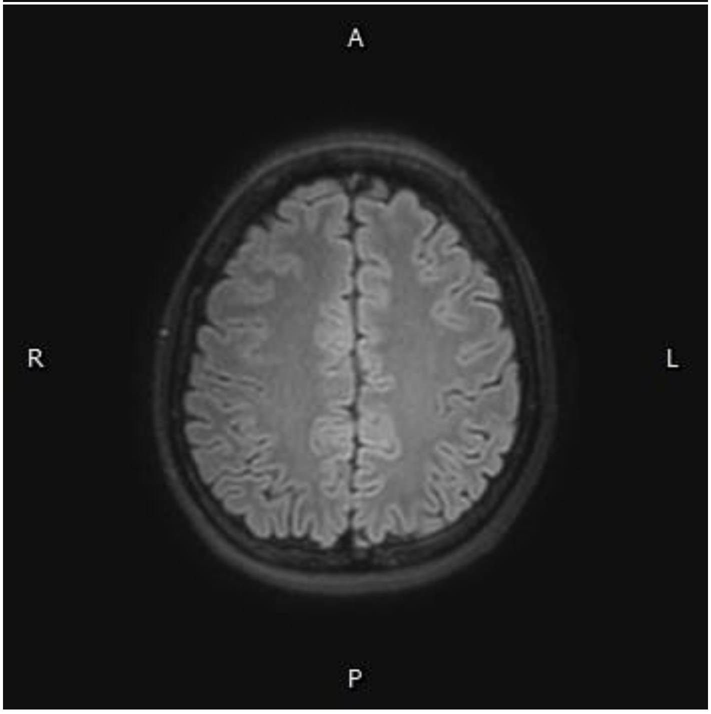
A
R
L
P

images of brain CT of Case 3 after AE onset

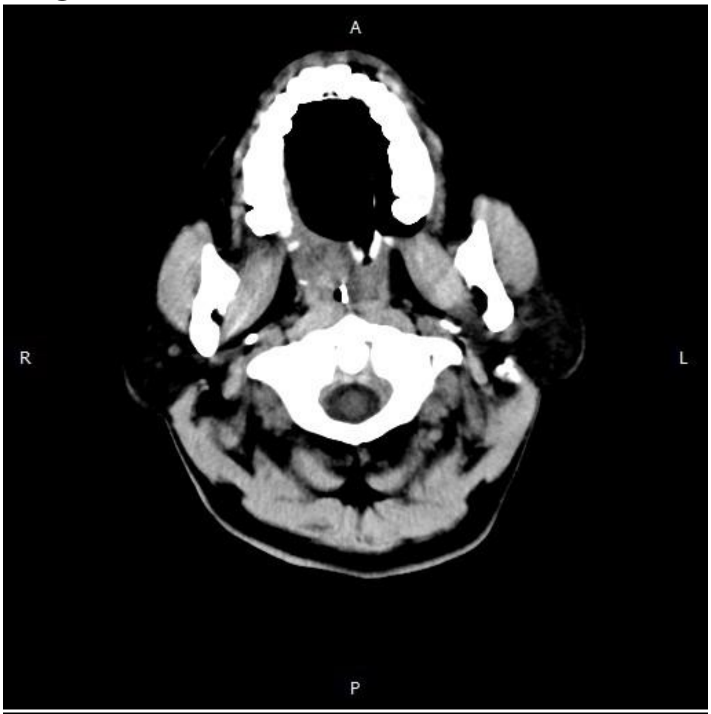

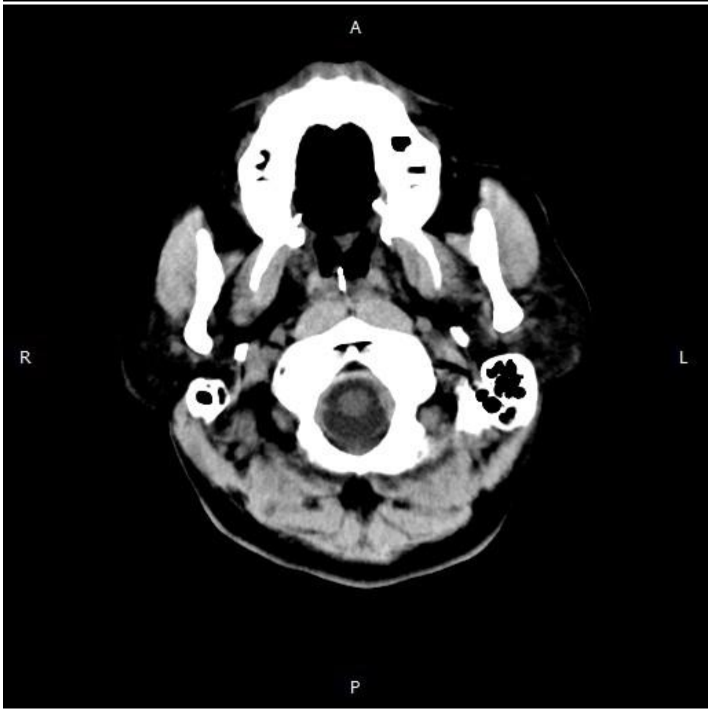

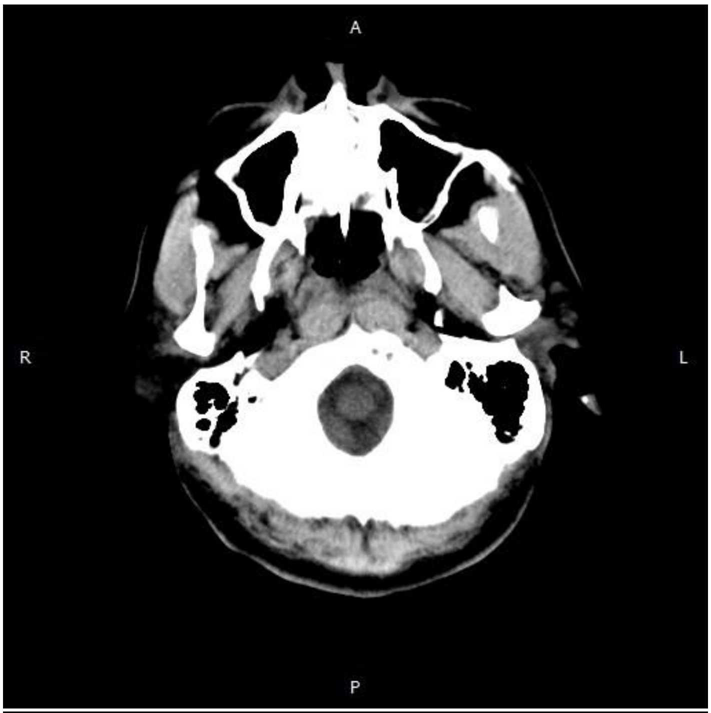
A
R
L
P
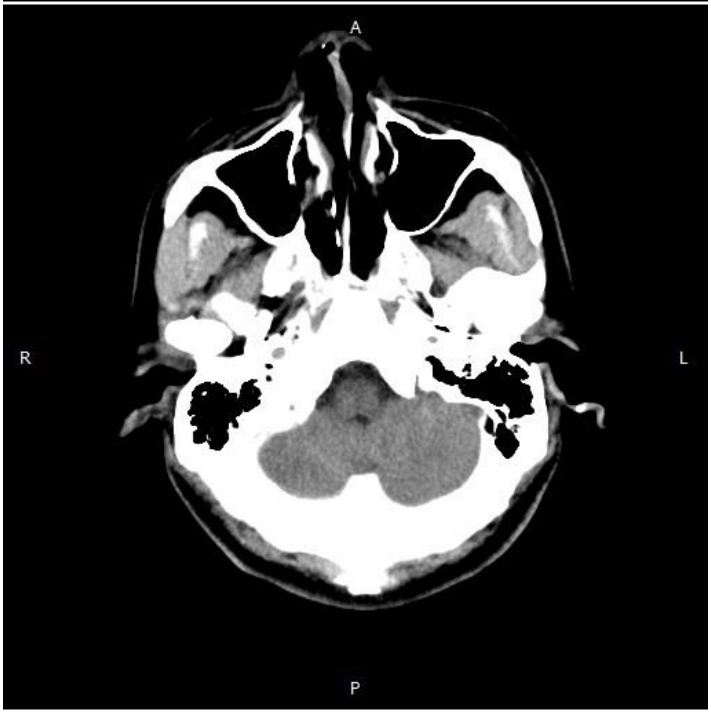
A
R
L
P

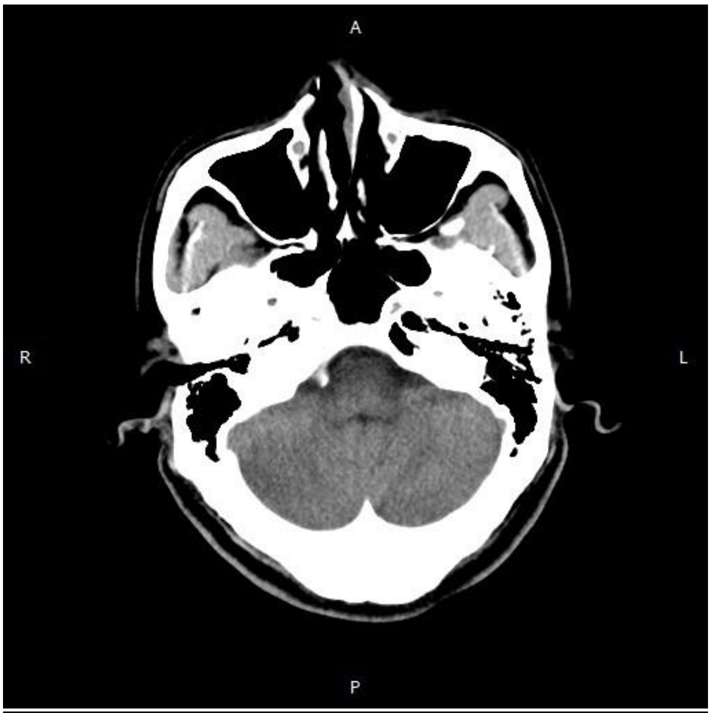
A
R
L
P

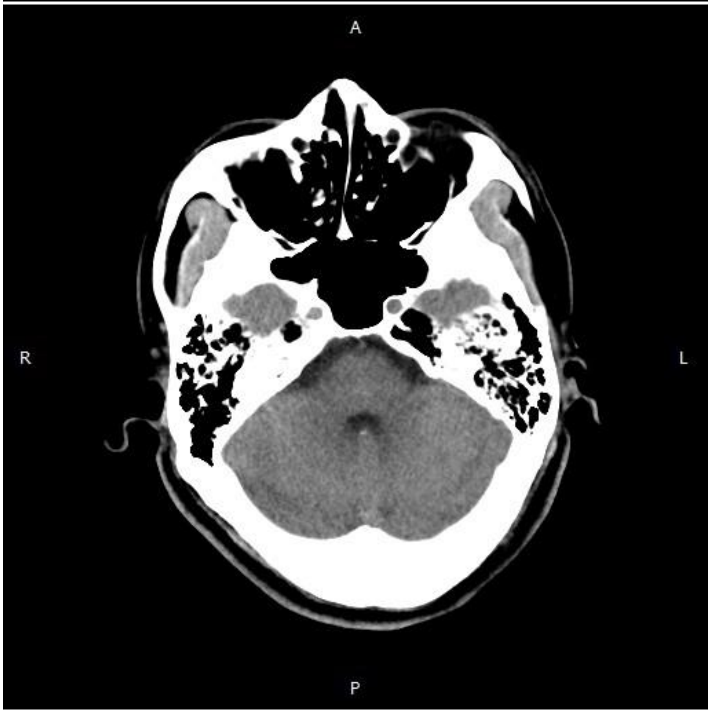
A
R
L
P

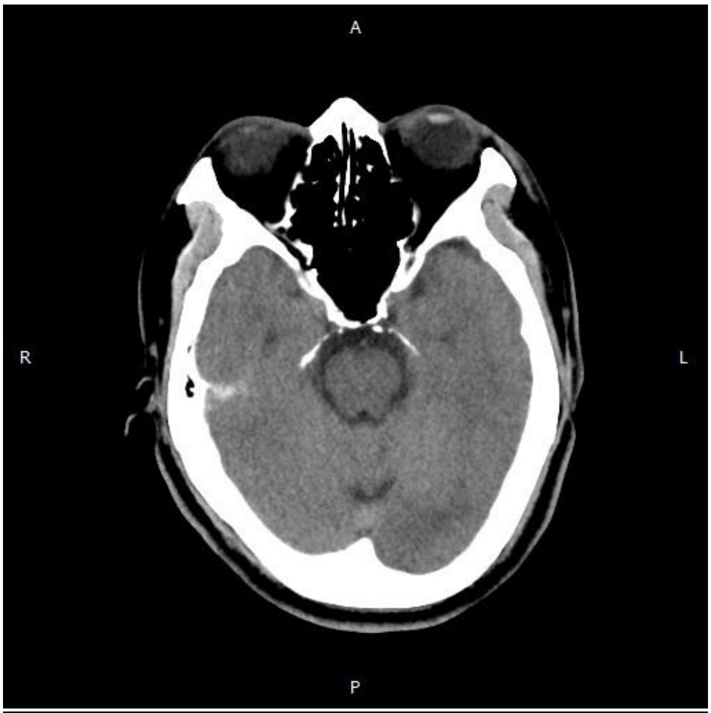
A
R
L
P

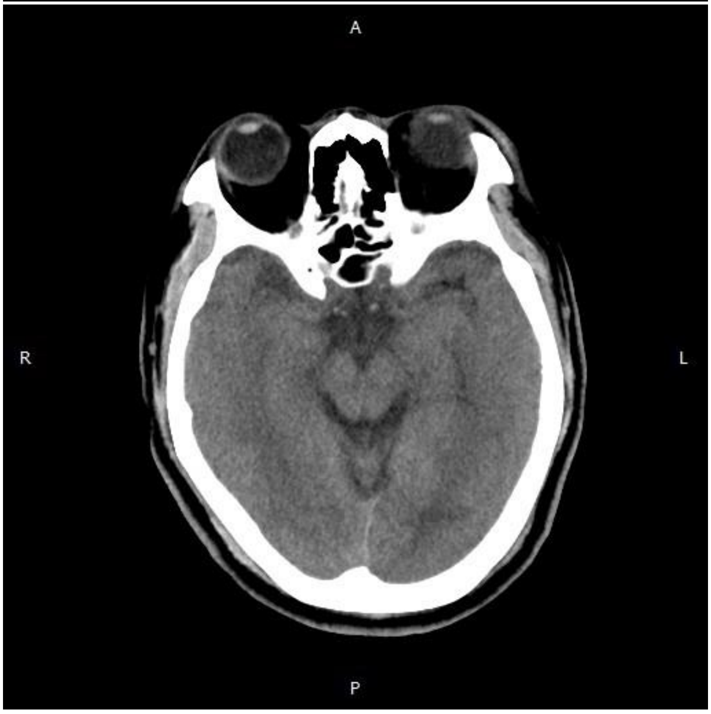
A
R
L
P

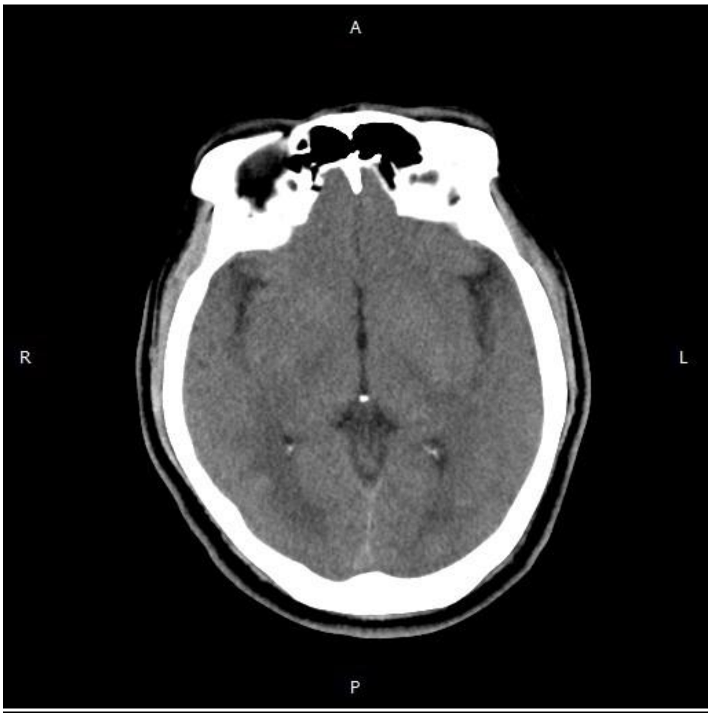
A
R
L
P

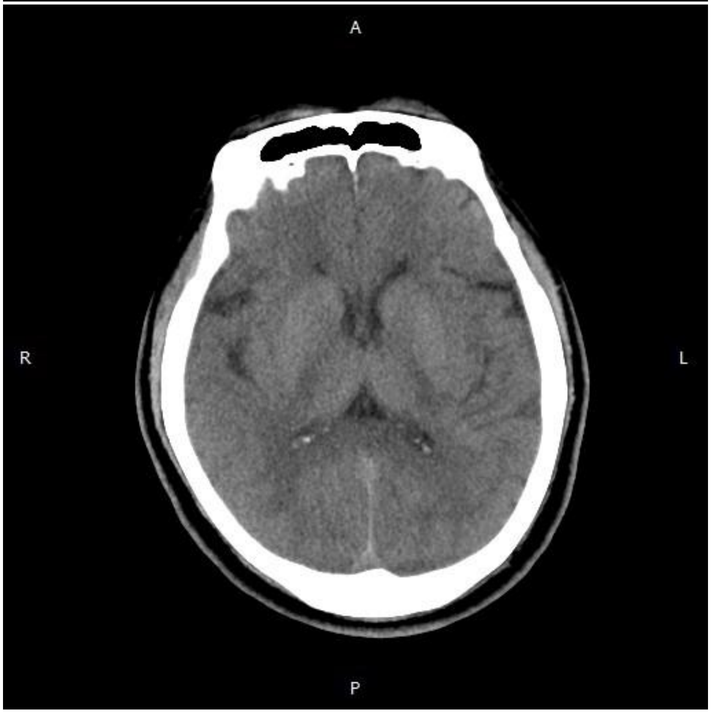
A
R
L
P

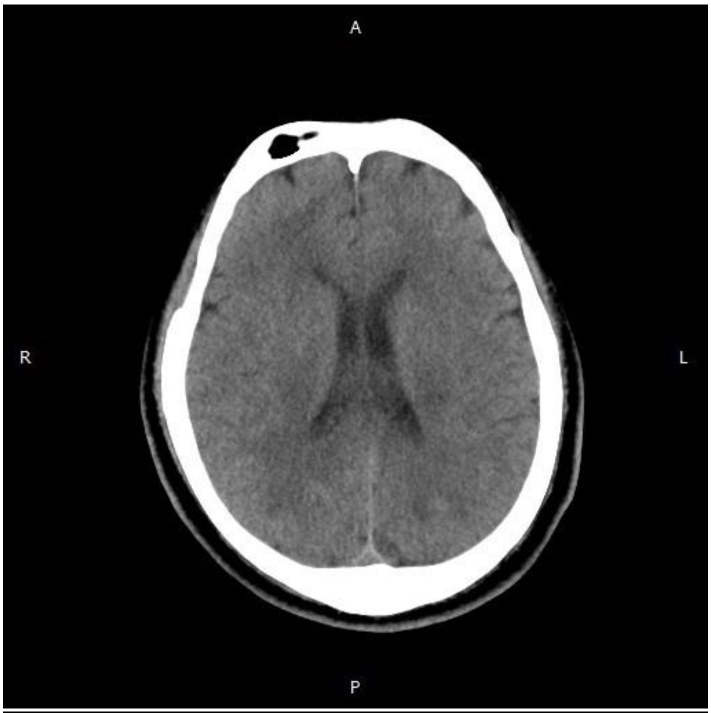
A
R
L
P

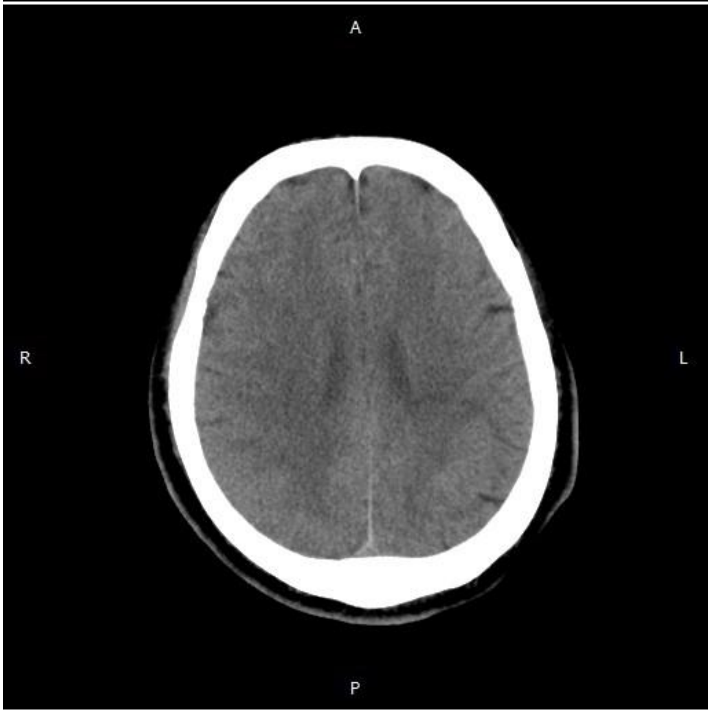
A
R
L
P

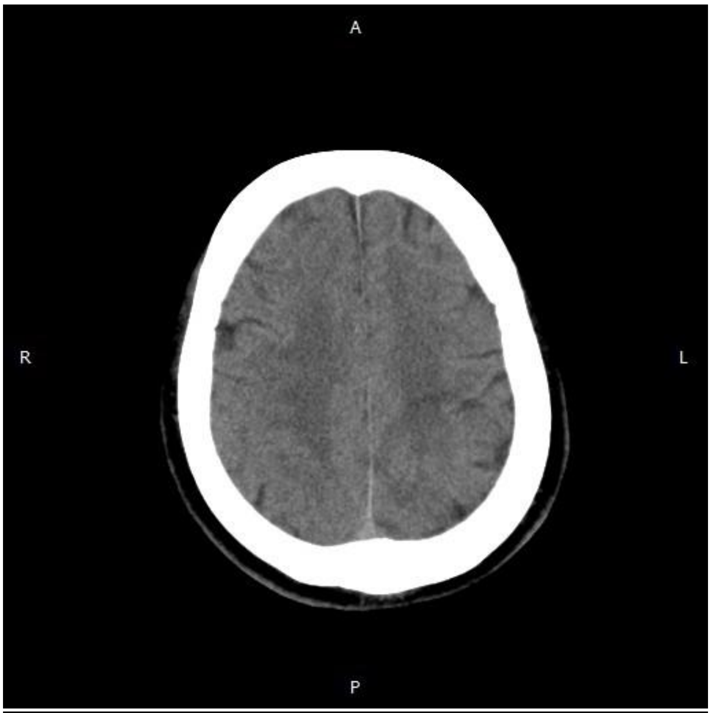
A
R
L
P

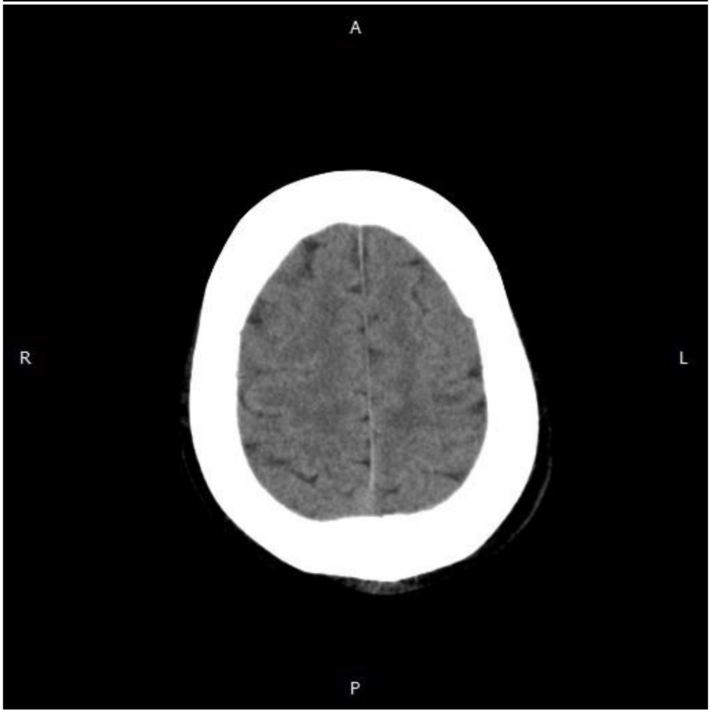
A
R
L
P

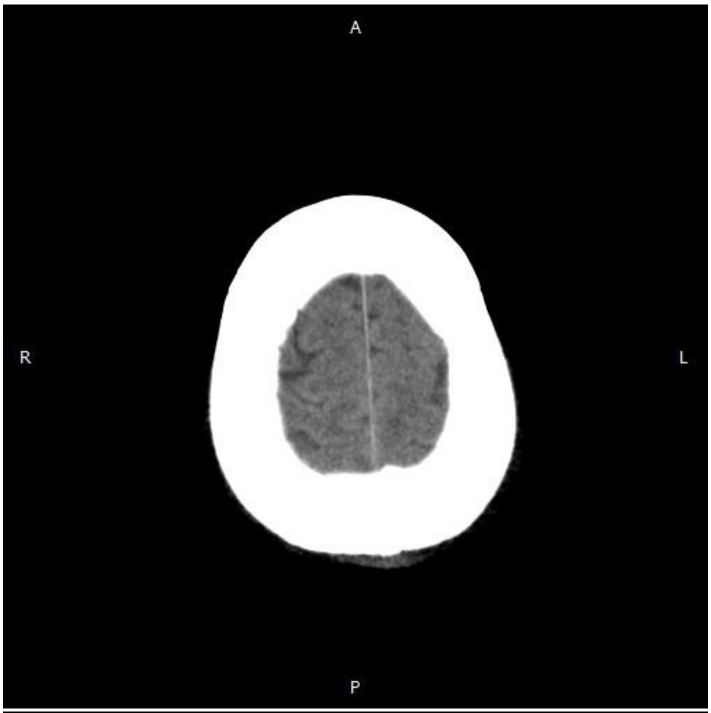
A
R
L
P

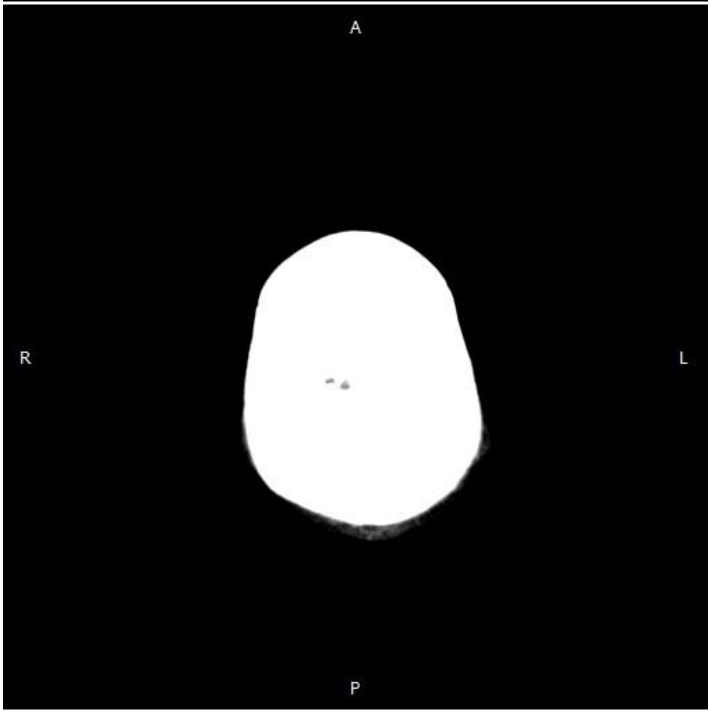
A
R
L
P

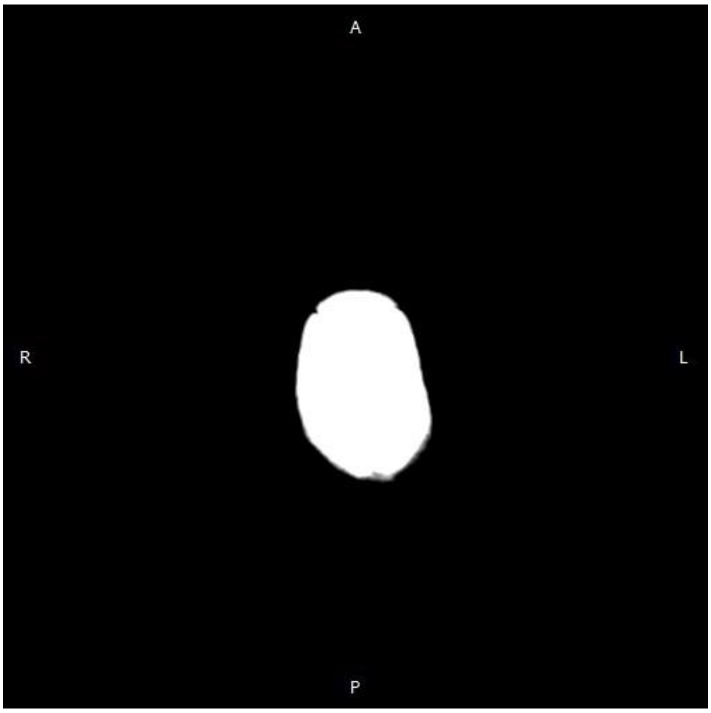
A
R
L
P
